# Evolution of malignant plasmacytoma cell lines from K14E7 Fancd2^−/−^ mouse long-term bone marrow cultures

**DOI:** 10.18632/oncotarget.12036

**Published:** 2016-09-15

**Authors:** Xichen Zhang, Wen Hou, Michael W. Epperly, Lora Rigatti, Hong Wang, Darcy Franicola, Aranee Sivanathan, Joel S. Greenberger

**Affiliations:** ^1^ Department of Radiation Oncology, University of Pittsburgh Cancer Institute, Pittsburgh, 15232 PA, USA; ^2^ Division of Laboratory Animal Resources, University of Pittsburgh, Pittsburgh, 15260 PA, USA

**Keywords:** E7, HPV oncogene, human papillomavirus, Fanconi anemia, Fancd2^−/−^ mice

## Abstract

We tested the effect of expression of the Human Papilloma Virus (HPV E7) oncogene on hematopoiesis in long-term bone marrow cultures (LTBMCs) derived from K14E7 (FVB) Fancd2^−/−^ (129/Sv), K14E7 Fancd2^+/+^, Fancd2^−/−^, and control (FVB X 129/Sv) Fl mice. K14E7 Fancd2^−/−^ and Fancd2^−/−^ LTBMCs showed decreased duration of production of total nonadherent hematopoietic cells and progenitors forming day 7 and day 14 multilineage CFU-GEMM colonies in secondary cultures (7 wks and 8 wks respectively) compared to cultures from K14E7 Fancd2^+/+^ (17 wks) or control mice (18 wks) *p* < 0.0001. Marrow stromal cell lines derived from both K14E7 Fancd2^−/−^ and Fancd2^−/−^ cultures were radiosensitive, as were IL-3 dependent hematopoietic progenitor cell lines derived from K14E7 Fancd2^−/−^ cultures. In contrast, Fancd2^−/−^ mouse hematopoietic progenitor cell lines and fresh marrow were radioresistant. K14E7 Fancd2^−/−^ mouse freshly explanted bone marrow expressed no detectable K14 or E7; however, LTBMCs produced K14 positive factor-independent (FI) clonal malignant plasmacytoma forming cell lines in which E7 was detected in the nucleus with p53 and Rb. Transfection of an E6/E7 plasmid into Fancd2^−/−^, but not control Fancd2^+/+^ IL-3 dependent hematopoietic progenitor cell lines, increased cloning efficiency, cell growth, and induced malignant cell lines. Therefore, the altered radiobiology of hematopoietic progenitor cells and malignant transformation *in vitro* by K14E7 expression in cells of the Fancd2^−/−^ genotype suggests a potential role of HPV in hematopoietic malignancies in FA patients.

## INTRODUCTION

Fanconi Anemia (FA) patients display a variety of inherited and acquired phenotypes [1], which are dependent upon mutation or deletion of one or more of the 18 gene products in the FA pathway [2–3]. Proteins in the FA complex form a scaffold onto which bind other proteins involved in the process of DNA repair and lead to monoubiquination of Fancd2, which when bound to FancI faciliates repair of DNA double strand breaks [4]. The FA pathway proteins are involved in other molecular mechanisms of disease including genome instability [4], tumorigenesis [5–6], hematopoietic failure [7], and radiosensitivity [8–10].

Human papillomavirus (HPV) is involved in genesis of squamous cell cancer of the head and neck, esophagus, and cervix [11–15]; however, its possible role in hematologic malignancies [16] has not been established. Fanconi Anemia (FA) patients have an increased frequency of squamous cell head and neck cancers [17–20]. The hypotheses that FA patients are more susceptible to malignant transformation of squamous cells by HPV, and that evolution of such cancers might relate to the intrinsic cellular radiosensitivity and FA pathway protein alterations, are subjects of current investigation [6, 17, 21–23]. Some HPV positive head and neck squamous cell cancers are radiosensitive [17]. HPV can alter DNA double strand break repair by homologous recombination [23], (a pathway which is already defective in FA patients [2–3]). HPV enhances TGF-β signaling [24], which may be deleterious in FA patients, who have a hyperactive response to TGF-β [25].

Recently, Fanconi Anemia (FA) Fancd2^−/−^ (129/Sv) mice with cytokeratin 14 (K14) promoter controlled production of E7 onco-protein of HPV derived from genetic cross with K14E7 mice (FVB/n) were shown to be susceptible to both chemical carcinogen induced oral squamous cell tumors, and estrogen pellet induced cervix cancers [26]. The data suggested that in the presence of a chemical carcinogen or estrogen stimulation, the E7 onco-protein interacted with an element(s) of the defective FA pathway in Fancd2^−/−^ mice and could induce malignant transformation of squamous epithelium in tissues that naturally express cytokeratin 14.

In the present studies, we determined whether culture of K14E7 Fancd2^−/−^ mouse marrow in LTBMCs [28] facilitated expression of K14 driven E7, and whether there was altered hematopoiesis. While K14E7 Fancd2^−/−^ mouse LTBMCs retained some Fancd2^−/−^ phenotypes including: suppressed duration of hematopoiesis and radiosensitivity of marrow stromal cell lines, we discovered that culture-derived hematopoietic cell lines, as well as fresh marrow hematopoietic progenitors were also radiosensitive, a distinct difference from Fancd2^−/−^ marrow. Furthermore, cytokeratin 14 and E7 were expressed in cultured K14E7 Fancd2^−/−^ marrow and nonadherent cells subcultured in Interleukin-3 (IL-3) generated factor-independent (FI) malignant plasmacytoma cell lines.

## RESULTS

### Reduced longevity of hematopoiesis in K14E7 fancd2^−/−^ mouse LTBMCs

**Figure 1:**
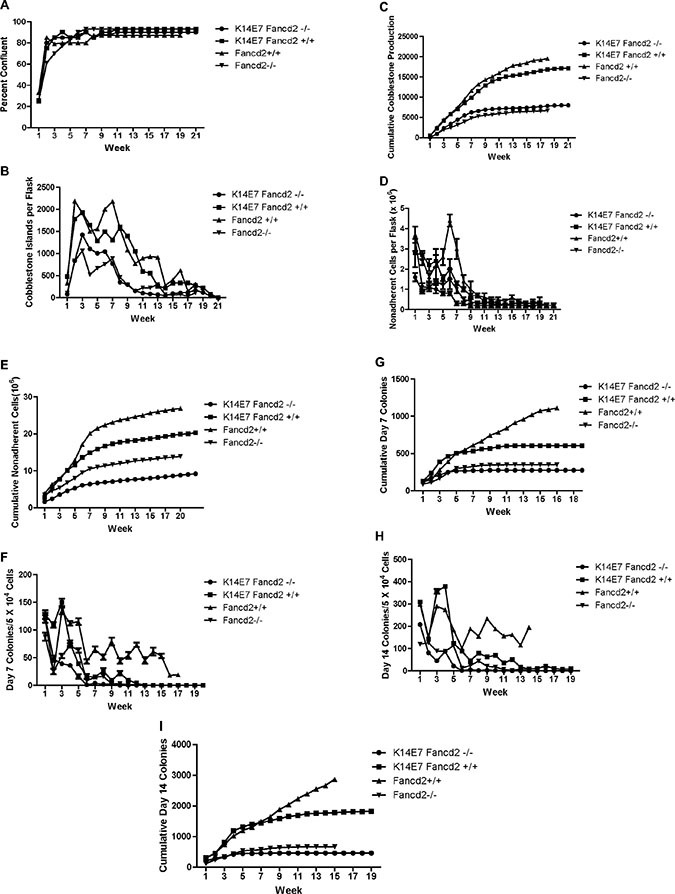
LTBMCs from K14E7Fancd2^−/−^ mice show reduced longevity of hematopoiesis. (**A**) adherent area confluent with stromal cells; (**B**) weekly count of cobblestone islands; (**C**) cumulative cobblestone islands; (**D**) weekly count of NA cells; (**E**) cumulative NA cells; (**F**) weekly scoring of D7 CFU-GEMM; (**G**) cumulative D7 CFU-GEMM; (**H**) weekly scoring of D14 CFU-GEMM; and (**I**) cumulative D14 CFU-GEMM. (*N* = 8 cultures/genotype)

Long-term bone marrow cultures were established from the tibia and femur marrow of four mice (8 cultures) of each genotype: K14E7Fancd2^−/−^, K14E7Fancd2^+/+^ (FVB/N), Fancd2^+/+^ (129/Sv), and control (129/Sv X FVB) F_1_ mice [26], and maintained according to published methods [9]. As shown in Figure 1A, adherent cell layers reached confluence within six weeks for all genotype groups. There was a slight delay (not statistically significant) (Supplementary Table S1) in the time to confluence of adherent cell layers in Fancd2^−/−^ mouse long-term bone marrow cultures. The cobblestone island numbers on the adherent layer of each flask (indicative of hematopoietic stem cell interaction with stromal cells of the hematopoietic microenvironment, and the robustness of hematopoiesis) are shown in Figure 1B (Supplementary Table S2). Marrow cultures from Fancd2^+/+^ and K14E7Fancd2^+/+^ mice showed greater numbers of cobblestone islands during weeks 1 through 14 compared to those detected in either K14E7Fancd2^−/−^ or Fancd2^−/−^ groups (Figure 1B). Cumulative cobblestone island production (Figure 1C) also showed greater numbers in cultures from the Fancd2^+/+^ genotypes. These results showing decreased production of hematopoietic cells in LTBMCs form K14E7 Fancd2^−/−^ mice confirm and extend those results of decreased hematopoietic cell production shown with both Fancd2^−/−^ (C57BL/6J) [9] and Fancd2^−/−^ (129/Sv) mouse LTBMCs [10].

The weekly production of non-adherent cells (Figure 1D) and cumulative cell numbers per flask (Figure 1E) were increased in cultures from Fancd2^+/+^ and K14E7 Fancd2^+/+^ mice (Supplementary Table S3). Results for cumulative production (Figure 1E) were also higher in cultures from Fancd2^+/+^ or K14E7Fancd2^+/+^ mice. In contrast, K14E7Fancd2^−/−^ and Fancd2^−/−^ mouse long-term bone marrow cultures showed decreased weekly (Figure 1D) and cumulative (Figure 1E) production of non-adherent cells. These results showing reduced overall culture longevity were similar to those with Fancd2^−/−^ C57BL/6 bone marrow cultures [9]. Therefore, the reduced longevity of hematopoiesis in LTBMCs derived from Fancd2^−/−^ mice of a different (129/Sv) genetic background was similar to that of Fancd2^−/−^ marrow from C57BL/6 mice [9]. The data also show that addition of the K14E7 genotype did not alter the reduced longevity of hematopoietic cell production in Fancd2^−/−^ marrow cultures.

The production of multilineage hematopoietic cells forming colonies in secondary culture (scored as those with greater than 50 cells at day 7 or day 14) were next evaluated. As shown in (Figure 1F, 1G) (Supplementary Table S4), weekly production of day 7 CFU-GEMM colonies, and also those cells forming colonies of greater and equal to 50 cells at 14 days (Figure 1H, 1I) (Supplementary Table S5) (indicative of more primitive hematopoietic cell progenitors) was clearly decreased in both Fancd2^−/−^ (129/Sv) and K14E7 Fancd2^−/−^ mouse long-term bone marrow cultures. The production of both day 7 and day 14 colony forming cells was decreased with respect to both weekly and cumulative production by K14E7Fancd2^−/−^ mouse marrow cultures.

Long-term cultures from K14E7Fancd2^+/+^ mice showed an earlier plateau with respect to cumulative cell production of day 7 (Figure 1G) and day 14 (Figure 1I) CFU-GEMM compared to the continuous production of hematopoietic cell colony forming units in Fancd2^+/+^ (129/Sv) marrow cultures. The effects of the K14E7 genotype on Fancd2^+/+^ long-term marrow culture production of day 14 colony forming cells, weekly (Figure 1H) and cumulative (Figure 1G) were as pronounced as that observed with day 7 colony forming cells. As shown in Figure 1H), there was a plateau in production of day 14 hematopoietic colony forming cells at around week 6 in K14E7Fancd2^+/+^ cultures, while numbers continued to increase weekly and in cumulative fashion for Fancd2^+/+^ marrow derived colony forming cells. These data establish that expression of K14E7 in Fancd2^−/−^ marrow did not alter the suppressed duration of hematopoiesis observed in Fancd2^−/−^mouse long-term bone marrow cultures (Supplementary Tables S1–S5).

### Long-term marrow cultures derived from oral 4-NQO treated K14E7Fancd2^−/−^ mice demonstrate no alteration in the duration of hematopoiesis

**Figure 2:**
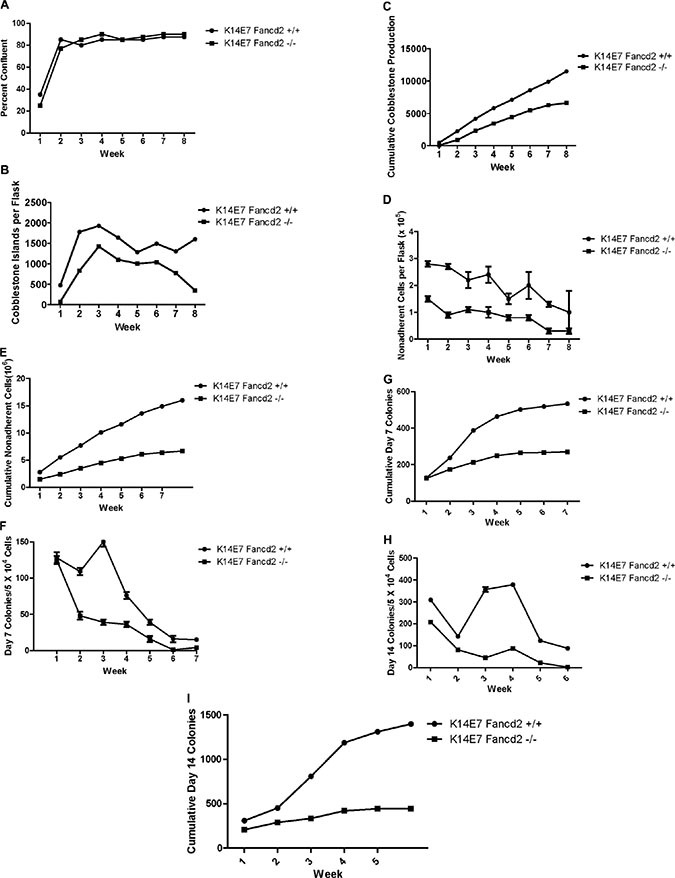
LTBMCs from oral-4NQO treated K14E7Fancd2^−/−^ mice show reduced duration of hematopoiesis. Groups of K14E7 Fancd2^+/+^ and K14E7 Fancd2^−/−^ mice received 4NQO in drinking water at 100 μM for 10 weeks as published (26), then were placed on regular drinking water for 22 weeks. Marrow cultures were then established as described in the methods. (**A**) areas of confluent stromal cells; (**B**) cobblestone islands weekly; (**C**) cobblestone islands cumulative; (**D**) weekly NA cells; (**E**) Cumulative NA cells; (**F**) weekly D7 CFU-GEMM; (**G**) cumulative D7 CFU-GEMM; (**H**) weekly D14 CFU-GEMM; and (**I**) cumulative D14 CFU-GEMM

**Figure 4:**
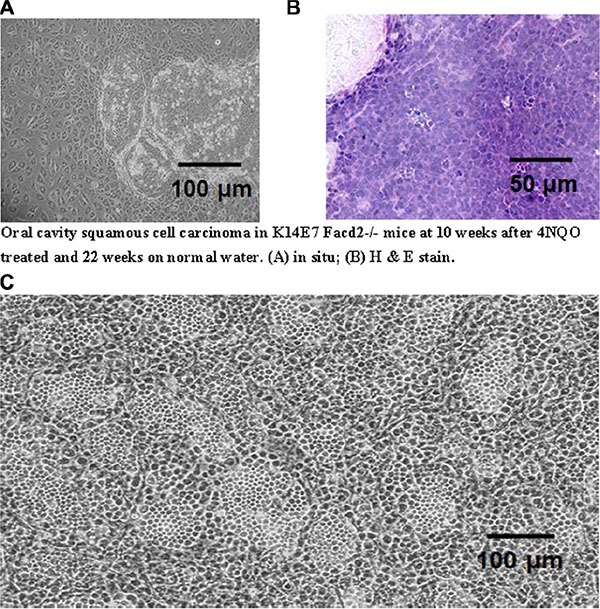
Comparison of morphology of explanted oral cavity tumor from a 4-NQO treated K14E7 Fancd2^−/−^ mouse with IL-3 cultured cells from LTBMCs. (**A**) Oral cavity tumor explanted in culture for 4 weeks; (**B**) original oral cavity tumor *in situ* (H & E stained); (**C**) cultured, clonal, nonadherent hematopoietic cells from K14E7 Fancd2^−/−^ mouse 12 weeks in 100 μM IL-3 after harvest from 4 wk old LTBMC

We next tested whether LTBMCs derived from 4-NQO treated K14E7Fancd2^−/−^ or K14E7Fancd2^+/+^ mice showed marrow toxicity or induced malignant transformation that was detectable in LTBMCs. Mice received 4-NQO in drinking water for the time-duration and under protocol conditions for induction of oral cavity cancers as described in the methods and in [26]. The K14E7 Fancd2^−/−^, but not K14E7 Fancd2^+/+^ mice in these experiments did develop oral cavity squamous cell tumors (Figure 4A–4B). Marrow from 4-NQO treated tumor-bearing K14E7 Fancd2^−/−^ or control 4-NQO treated K14E7 Fancd2^+/+^ mice was placed into LTBMC. As shown in Figure 2, there was no detectable effect of 4-NQO treatment on the genotype dependent time to reach a plateau in the confluence of the adherent layer of long-term marrow cultures (Figure 2A). K14E7Fancd2^−/−^ mouse marrow showed a decrease in weekly production and cumulative production of cobblestone islands (Figure 2B and 2C, respectively) similar to the data with LTBMCs from non 4-NQO treated mice (Figure 1). The results establish that reduced hematopoiesis in LTBMCs from K14E7 Fancd2^−/−^ mice was independent of previous oral 4-NQO treatment. In explanted marrow from mice that received 4-NQO in the drinking water, the production of non-adherent cells was decreased in K14E7Fancd2^−/−^ mouse compared to K14E7 Fancd2^+/+^ mouse marrow cultures measured weekly (Figure 2D) or by calculation of cumulative cell production (Figure 2E).

The production of nonadherent day 7 colony forming cells by 4-NQO treated K14E7 Fancd2^−/−^ compared to 4-NQO treated control K14E7 Fancd2^+/+^ mouse bone marrow cultures showed that former had a reduction in weekly (Figure 2F) and cumulative colony forming cell numbers (Figure 2G). Numbers of day 14 colony forming cells measured on a weekly (Figure 2H) and cumulative basis (Figure 2I) showed reduced numbers in K14E7 Fancd2^−/−^ cultures. These results establish that marrow from orally 4-NQO treated K14E7 Fancd2^−/−^ mice did not reveal overall alteration in *in vitro* hematopoiesis in LTBMCs nor did it increase or further suppress of the duration of hematopoiesis. There was some reduction in colony forming cells in 4-NQO treated K14E7 Fancd2^−/−^ cultures. There was also an increase in cell numbers in K14E7 Fancd2^+/+^ cultures at early weeks. Furthermore, the oral chemical carcinogen 4-NQO treatment did not stimulate detectable morphologic or phenotypic changes in LTBMCs held for 8 weeks after marrow explant.

### Radiosensitivity of LTBMC-derived stromal and hematopoietic progenitor cell lines and fresh marrow hematopoietic colony forming cells from K14E7 Fancd2^−/−^ mice

**Figure 3:**
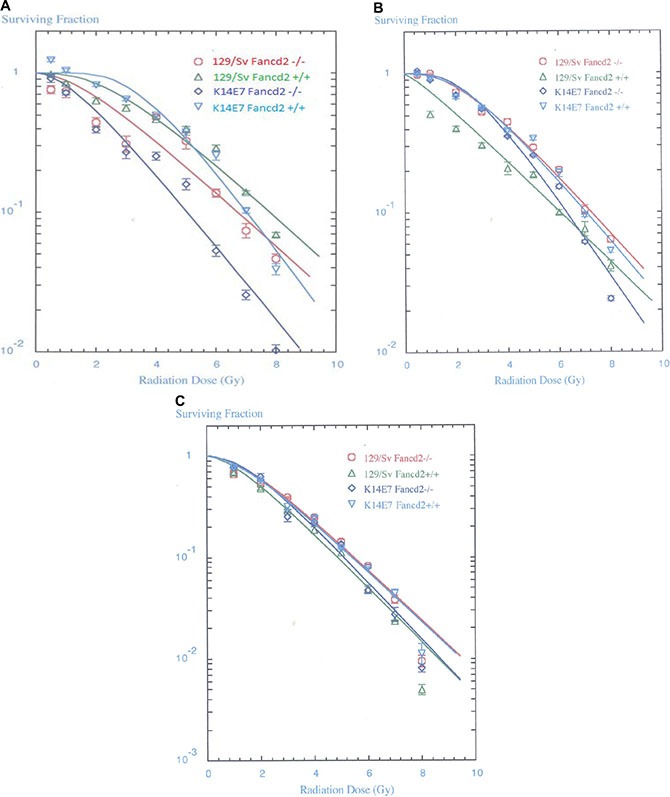
Radiosensitivity of clonogenic K14E7Fancd2^−/−^ bone marrow stromal and IL-3 dependent hematopoietic progenitor cell lines and fresh bone marrow. (**A**) Bone marrow stromal cell lines were established from the adherent layers of 4 week old LTBMCs. Clonal lines were isolated from long term bone marrow cultures established from groups of (FVB/N) K14E7 Fancd2^+/+^, (129/Sv) Fancd2^−/−^, (129/Sv) Fand2^+/+^, and K14E7Fancd2^−/−^ mice. Stromal cell lines were established according to published methods [9]. The cell lines were irradiated to doses ranging from 0 to 8 Gy, plated in 4 well Linbro plates, incubated for 7 days at 37°C, stained with crystal violet, and colonies of greater than 50 cells were counted (Table 1). (**B**) Radiation sensitivity of IL-3 dependent cell lines was determined as published [9] (Table 2). (**C**) Fresh bone marrow was isolated from 6–8 wk old female K14E7 Fancd2^+/+^, K14E7 Fancd2^−/−^, 129/Sv Fancd2^+/+^, and 129/Sv Fancd2^−/−^ mice. The CFU-GEMM assay scored at day 14 was carried out as described in the methods (Table 3). The data was analyzed using single-hit, multi-target models and linear quadratic models

We established clonal bone marrow stromal and hematopoietic progenitor cell lines from long-term marrow cultures of each mouse genotype. Clonal marrow stromal cell lines derived from K14E7 Fancd2^−/−^, as well as Fancd2^−/−^ mouse marrow cultures were radiosensitive (Figure 3A, Table 1). In contrast, clonal IL-3 dependent hematopoietic progenitor cell lines derived from Fancd2^−/−^ (129/Sv) LTMBCs (Figure 3B, Table 2), as well as fresh marrow colony forming progenitors (Figure 3C, Table 3) were radioresistant when scored for formation of 50 cell CFU-GEMM colonies. The results with Fancd2^−/−^ (129/Sv) marrow derived cell lines and fresh marrow CFU-GEMM [3] showing radioresistance of hematopoietic cells and radiosensitivity of stromal cells confirm and extend prior results showing the same patterns with hematopoietic compared to marrow stromal cell lines from Fancd2^−/−^ (C57BL/6) mice [9].

**Table 1:** Radiosensitivity of K14E7Fancd2^−/−^ marrow stromal cell lines

Cell Line	Do (GY)	ň
*K14E7 Fancd2*^+/+^	1.48 ± 0.05	4.7 ± 0.3
*K14E7 Fancd2*^−/−^	1.52 ± 0.15 P1 = 0.84	1.5 ± 0.5 P1 = 0.01
*129/Sv Fancd2*^+/+^	2.33 +0.11 P2 = 0.004	3.5 ± 0.1 P2 = 0.17
*129/Sv Fancd2*^−/−^	2.23 + 0.01 P3 = 0.4970 P4 = 0.036	1.9 ± 0.3 P3 = 0.030 P4 = 0.64

P1 compares the *K14E7 Fancd2*^−/−^ cell line to the *K14E7 Fancd2*^+/+^ cell line.

P2 compares the *129/Sv Fancd2*^+/+^ cell line to *K14E7 Fancd2*^+/+^ cell line.

P3 compares *129/Sv Fancd2*^−/−^ cell line to *129/Sv Fancd2*^+/+^ cell line.

P4 compares *129/Sv Fancd2*^−/−^ cell line to *K14E7 Fancd2*^−/−^ cell line.

P less than 0.05 shown in red.

Both *Fancd2*^−/−^ cell lines are more radiosensitive than their respective *Fancd2*^+/+^cell lines as seen by the decreased ň which represents the shoulder on the survival curve (P1 and P3). Both of the *K14E7 Fancd2* cell lines are more radiosensitive that their respective *SV129 Fancd2* cell lines as seen the decreased D_o_ (P2 and P4).

**Table 2:** Radiosensitivity of K14E7 Fancd2^−/−^ IL-3 dependent marrow culture-derived hematopoietic progenitor cell lines

Cell Lines	D_0_(Gy)	ñ
**K14E7 Fancd2^+/+^**	1.86 ± 0.06	5.4 ± 1.7
**K14E7 Fancd2^−/−^**	1.37 ± 0.12 (*p* = 0.025)	4.3 ± 1.1
**129/Sv Fancd2^+/+^**	1.08 ± 0.06	2.1 ± 0.05
**129/Sv Fancd2^−/−^**	2.02 ± 0.11 (*p* = 0.0017)	3.7 ± 0.07 (*p* = 0.027)

*p*-value compares K14E7 Fancd2^−/−^ to K14E7 Fancd2^+/+^ or 129/Sv Fancd2^−/−^ with 129/Sv Fancd2^+/+.^

**Table 3:** Radiosensitivity of K14E7 Fancd2^−/−^mouse fresh bone marrow CFU-GEMM

Fresh Marrow Source	Do (Gy)	ñ
K14E7 Fancd2^+/+^	1.84 ± 0.04 (*p* = 0.075)	5.6 ±2.8
K14E7 Fancd2^−/−^	1.47 ± 0.07 (*p* = 0.039)	2.5 ±0.3
129/Sv Fancd2^+/+^	1.47 ±0.15	3.5 ±1.5
129/Sv Fancd2^−/−^	1.85 ± 0.06 (*p* = 0.0223)	2.3 ±0.1

*p* value compares K14E7 Fancd2^−/−^ to K14E7 Fancd2^+/+^ or 129/Sv Fancd2^−/−^ to 129/Sv Fancd2^+/+^.

In marked contrast, K14E7 Fancd2^−/−^ LTBMC culture-derived clonal IL-3 dependent cell lines (Figure 3B) and fresh marrow derived CFU-GEMM (Figure 3C) were radiosensitive. Compared to results with Fancd2^−/−^ mice, freshly removed marrow and LTBMC-derived IL-3 dependent hematopoietic progenitor cell lines, from K14E7 Fancd2^−/−^ mice showed a clear change in phenotype in that hematopoietic cells, as well as bone marrow stromal cell lines were radiosensitive.

### Evolution of clonal malignant plasmacytoma forming cell lines from K14E7 Fancd2^−/−^ LTBMCs

The morphology of 4-NQO induced oral tumors in K14E7 Fancd2^−/−^ mice (Figure 4A–4B) was similar to that reported previously [26]. Unexpectedly, K14E7Fancd2^−/−^ LTBMC derived IL-3 dependent non-adherent cells harvested at either week 4 or week 14 (kept as both uncloned lines, and clonal lines expanded from single cell cultures and passaged weekly) produced both adherent and nonadherent cells in IL-3 supplemented secondary cultures (Figure 4C). Weekly passage of nonadherent cells continued to generate persistent adherent and nonadherent hematopoietic cells.

The observation of morphologic changes in K14E7 Fancd2^−/−^ marrow cells in IL-3 containing secondary culture suggested either the persistence of two cell populations (both adherent and nonadherent) or that these subcultures contained a novel bilineage mesenchymal/hematopoietic cell phenotype. To distinguish between these possibilities, clonal cell lines were derived from IL-3 dependent cell lines from LTBMCs of each mouse genotype. All clonal cell lines derived from K14E7 Fancd2^−/−^ marrow cultures showed the same pattern of both adherent and nonadherent cells (Figure 4C). In contrast, IL-3 dependent clonal cell lines derived from all other groups of LTBMCs showed only nonadherent suspension cultured cells. The cloning efficiency of IL-3 dependent cells in subcultures from K14E7 Fancd2^−/−^ LTBMCs was significantly higher (19/20 single cells) than those obtained from K14E7 Fancd2^+/+^ IL-3 dependent secondary cell cultures (1/50) single cells.

**Figure 5:**
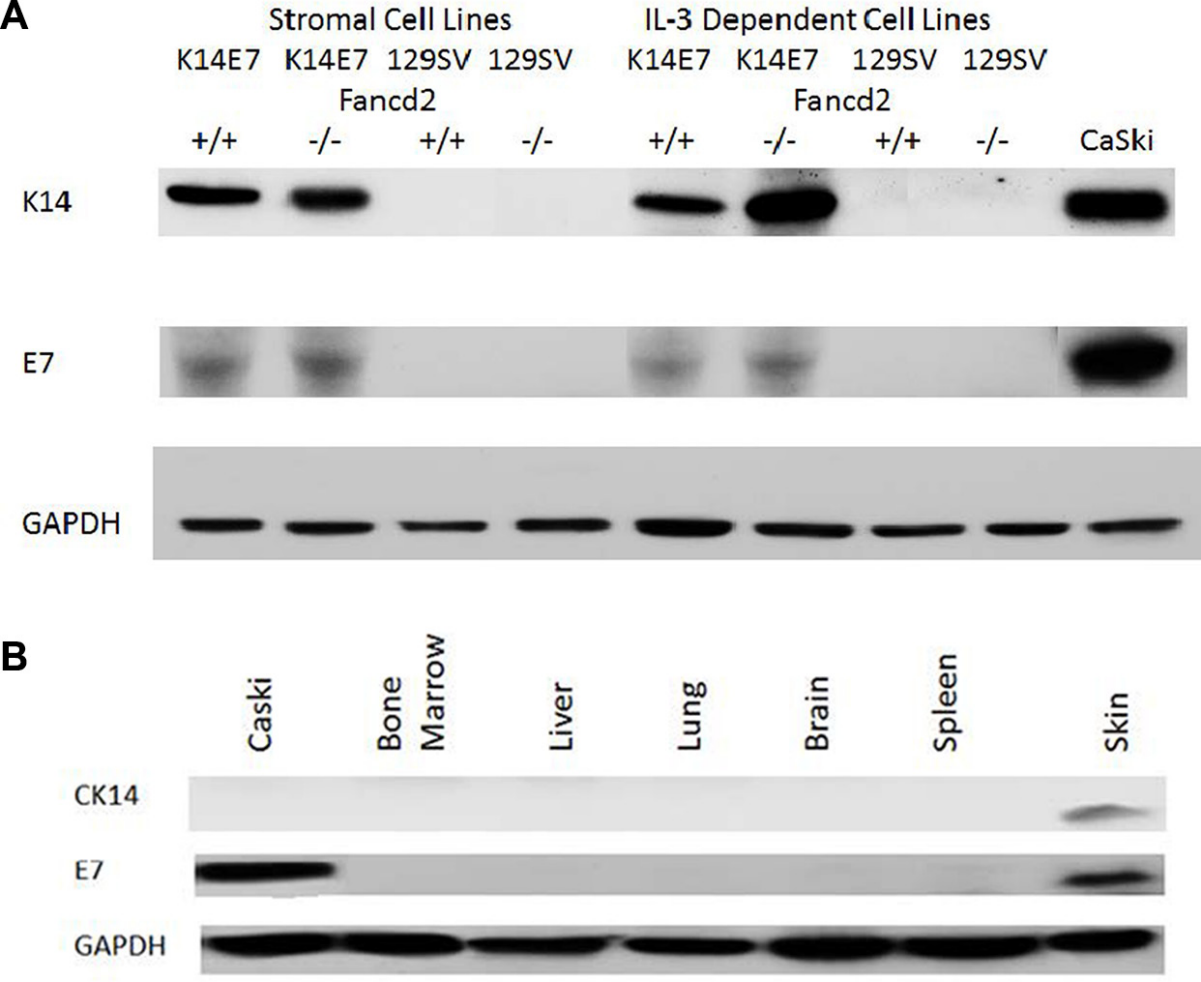
Detection of K14 and E7 proteins by Western blot in fresh tissues and in bone marrow stromal and IL-3 dependent cell lines derived from K14E7Fancd2^−/−^ mice LTBMCs. (**A**) Cell lines of bone marrow stromal compared to IL-3-dependent cell lines; and (**B**) Fresh explanted tissues (100 μM) for 4 weeks

**Figure 6:**
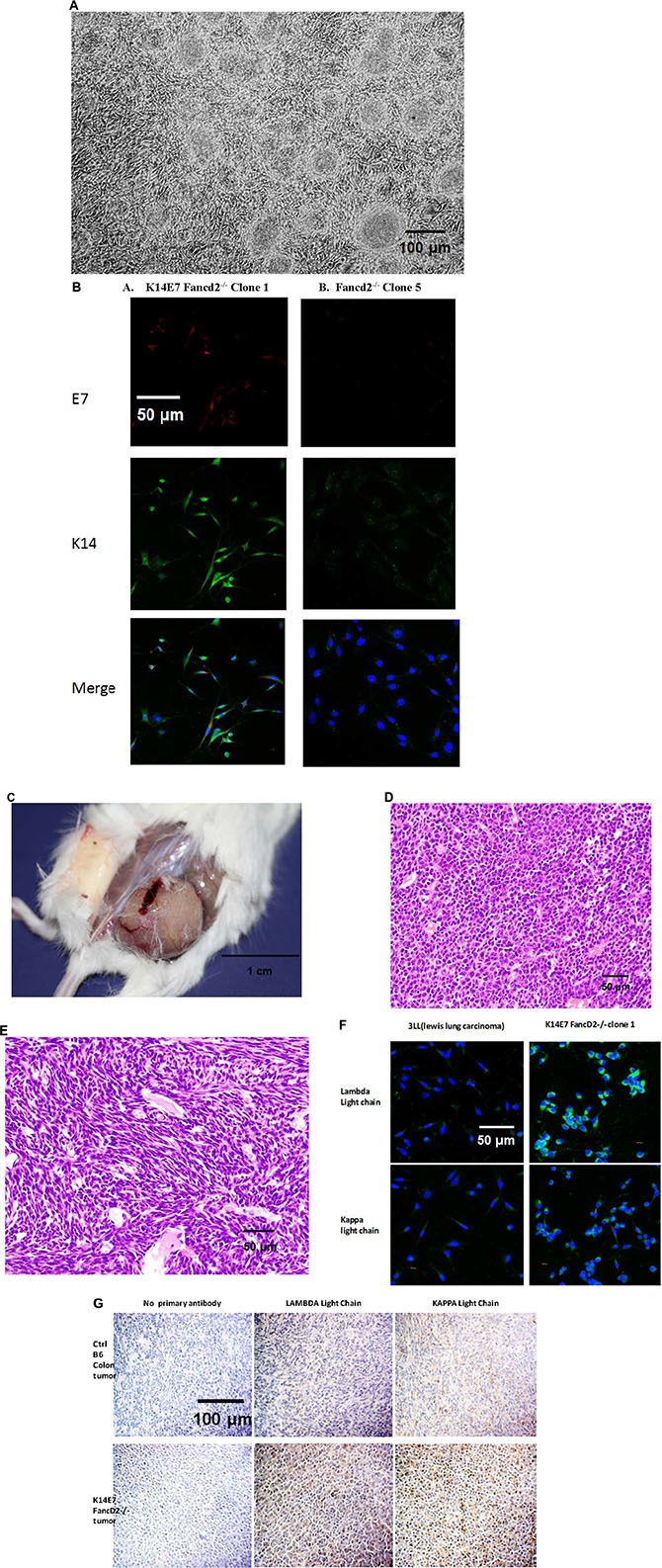
Morphology and immunohistochemistry for immunoglobulin kappa and lambda light chain in K14E7 Fancd2^−/−^ clone 1 IL-3-dependent tumor forming cell lines. (**A**) *in vitro* appearance of FI (no IL-3) clonal cell line K14E7 Fancd2^−/−^ clone 1; (**B**) cytokeratin-14 (K14–green) and E7 (red) positivity of K14E7 Fancd2^−/−^ clone 1, Blue nucleus–DAPI-stain compared to control Fancd2^−/−^ clone 5; (**C**) tumor formed in flank of mouse at wk 4 after injection with 10^6^ K14E7 Fancd2^−/−^ clone 1 cells; and (**D**) H&E stained tumor-plasmacytoma appearing area from tumor in panel C; and **E**) sarcoma appearing area in same tumor as in panel (D). (**F**) Immunoglobulin K and B light chain positivity of cells from K14E7 Fancd2^−/−^ clone 1 cell line; (**G**) immunoglobulin light chain kappa and lambda positive tumor induced *in vivo* by K14E7 Fancd2^−/−^ clone 1 cell line (tumor from panel C) negative control Lewis Lung Carcinoma (3LL) cell line [67]

We next tested K14E7 Fancd2^−/−^ adherent stromal and the novel IL-3 dependent cell lines for expression of K14 and E7 and compared this data with that from freshly removed organs from the same K14E7 Fancd2^−/−^, as well as control K14E7 Fancd2^+/+^, and other control mice. As shown in Figure 5A, the adherent stromal cell lines from both K14E7 Fancd2^+/+^ and K14E7 Fancd2^−/−^ LTBMCs were positive for both K14 and E7, while Fancd2^−/−^ and 129/Sv cell lines were negative (Supplementary Table S6). Freshly removed epithelial squamous cell containing organs from K14E7 Fancd2^−/−^ mice including: skin, oral cavity, esophagus, and cervix were positive for both K14 and E7; however, other freshly removed organs including bone marrow were negative (Figure 5B). Furthermore, K14 and E7 negative tissues (other than marrow) cultured in medium supplemented with IL-3 for 4 weeks remained negative for detectable K14 and E7 (data not shown). Clonal K14E7 Fancd2^−/−^ cell lines grown in IL-3 were next tested for IL-3 independence, and all were IL-3 independent, factor-independent (F1) cell lines. In contrast, clonal K14E7 Fancd2^+/+^, (129/Sv X FVB/n) F1 and Fancd2^−/−^ cell lines remained IL-3 dependent. The K14E7 Fancd2^−/−^ clonal lines grew densely in culture (Figure 6A), and were positive for both K14 and E7 (Figure 6B).

We next injected K14E7 Fancd2^−/−^ clonal FI and other IL-3 dependent cell lines subcutaneously into the flank of adult mice at 1 × 10^6^ cells in 100 μl medium. Only the K14E7 Fancd2^−/−^ FI cell lines formed tumors (Figure 6C, Table 4). The tumor histopathology showed both plasmacytoma (Figure 6D) and spindle cell sarcoma-like (Figure 6E) regions. Cells from the same K14E7 Fancd2^−/−^ clone 1 cell line injected I.V. at 1 × 10^6^ cells in 100 μl formed multiple lung, liver, and soft tissue metastases, replaced the bone marrow with tumor cells, and produced peripheral blood leukemia with Ig kappa and lambda positive cells (not shown). The tumor-inducing K14E7 Fancd2^−/−^ clone 1 cell line (Figure 6F) and the tumors induced by this cell line (Figure 6G) were positive for both immunoglobulin kappa and lambda light chains, consistent with a plasma cell tumor. Lambda and kappa were upregulated and the ratio of lambda to kappa was increased. Since the K14E7 Fancd2^−/−^ clone1 derived tumor cell line was single cell in origin, the results establish that both kappa and lambda light chains, as well as the sarcoma morphology were found in the same clonal tumors. In contrast, K14E7 Fancd2^+/+^ adherent or IL-3 dependent nonadherent cell lines injected S.C. or I.V. produced no leukemia or solid tumors (not shown). These results establish that the E7 oncogene of HPV induced malignant transformation of bone marrow from K14E7 Fancd2^−/−^ mice when grown in LTBMCs *in vitro*.

**Table 4:** Tumor formation by K14E7 Fancd2^−/−^bone marrow culture derived cell lines

± 13 Dependent Cell Line	Morphology in Culture	No. Cells Injected	No. Tumors/No. Mice	%
Fancd2^−/−^(129/Sv)	H	10^7^	0/10	0
K14E7 (FVB) Fancd2^+/+^(129/Sv)	H	10^7^	0/10	0
K14E7 Fancd2^−/−^	B	10^7^	1/5	60
K14E7 Fancd2^−/−^Clone 1	B	10^7^	3/5	20
K14E7 Fancd2^−/−^Clone 5	B	10^7^	2/4	50
K14E7 Fancd2^−/−^Clone 6	B	10^6^	3/5	60
K14E7 Fancd2^−/−^Clone 8	B	10^6^	1/5	20

H = Hematopoietic Nonadherent.

B = Bilineage Adherent and Nonadherent Cells, as in Figure 4C.

### Novel phenotype of K14E7 Fancd2^−/−^ LTBMC derived mouse plasmacytoma inducing cell lines

The cell surface phenotype and immunohistochemical profile of the K14E7 Fancd2^−/−^, IL-3 dependent, (which rapidly became IL-3 independent tumorigenic clonal cell lines (clone 1)) was markedly different from that observed with Fancd2^−/−^ (129/Sv) IL-3 dependent cell line. There was an increase in SCA1, from 2.3 to 97.5% positive. Collagen IV decreased from 27.7 to 0.5% (Table 5). The tumor-inducing K14E7 Fancd2^−/−^, cell line had low levels of some markers of mesenchymal cells including osteoblasts (osteocalcin) and endothelial/sarcoma (Collagen IV) (Table 5).

**Table 5:** Immunohistochemical phenotype of tumorigenic cell line K14E7 Fancd2^−/−^ clone 1

Antibody	Known Cell Type Positive	K14E7 Fancd2^−/−^ Clone 1 (% positive)	Fancd2^−/−^(129/Sv) IL-3 dependent cell line (% positive)
CD3e	NK, NKT, T, Th17, and Treg cells	26.4	0.3
CD5	B and T cells	29.0	1.5
CD8	NK, NKT, and T cells	43.5	0.8
B220	B and T cells	32.4	1.0
GR-1	Macrophage and Monocyte cells	17.5	0.0
TER-119	Erythroid cells	11.8	0.1
CD41	Hematopoietic Stem cells	0.1	0.0
CD45	Hematopoietic cells except erythrocytes and platelets	17.2	100.0
CD48	NK, NKT, and T cells	7.3	14.0
SCA-1	Hematopoietic Progenitor/Stem cells	97.5	2.3
C-KIT	Hematopoietic Progenitor/Stem cells	99.6	100.0
CD150	B, T, Dendritic cells	7.3	1.1
Vimentin	Fibroblasts	54.8	51.1
Actin	Muscle	4.5	55.6
Adiponectin	Adipocytes and Pre-Adipocytes	7.0	8.3
Collagen III	Fibroblasts	10.3	18.8
Collagen IV	Endothelial cells	0.5	27.7
Osteopontin	Osteoblasts	12.1	3.6
Osteocalcin	Osteoblasts	0.2	0.6

### Detection of E7, p53 and Rb in the nucleus of stromal and hematopoietic cell lines

**Figure 7:**
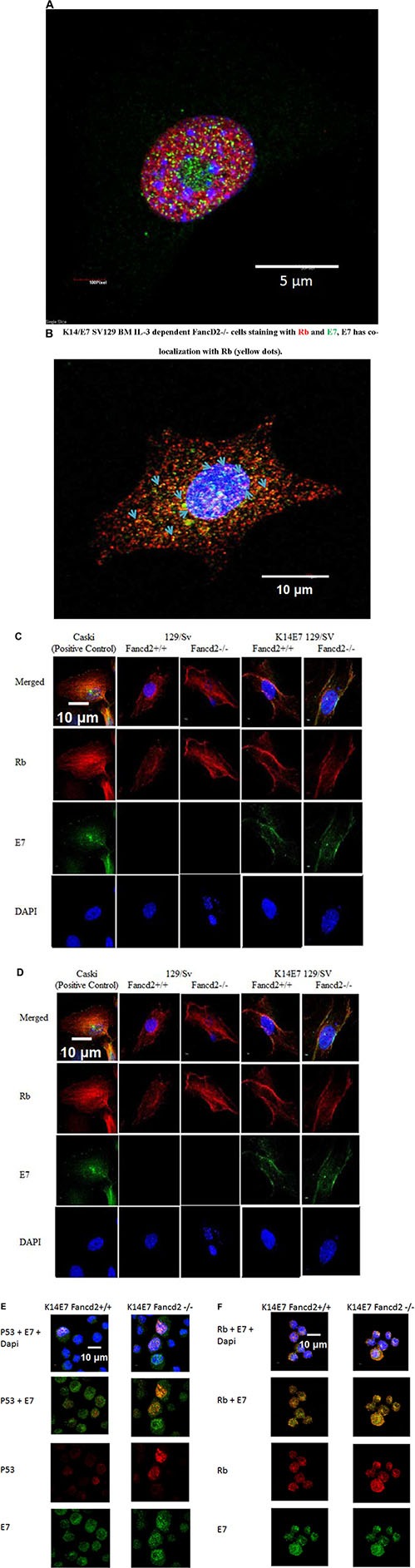
Detection of HPV E7 with p53 and Rb in the nucleus and cytoplasm of K14E7 Fancd2^−/−^ stromal and hematopoietic cell lines. (**A**) E7 and p53 detection in the nucleus of K14E7 Fancd2^−/−^ clone 1 cells (nucleolus is green). Detection of E7 with p53 (yellow dots); (**B**) E7 and Rb detection in the nucleus and cytoplasm of K14E7 Fancd2^−/−^ clone 1 cells (nucleus is blue). E7 detected with Rb (yellow dots).; (**C**) p53 with E7 in stromal cell lines (× 500); (**D**) Rb with E7 in stromal cell lines; (**E**) p53 with E7 in IL-3 dependent hematopoietic cell line; (**F**) Rb with E7 in IL-3 dependent hematopoietic cell lines

We tested K14E7 Fancd2^−/−^ malignant clonal lines, tumor cell lines removed form sites of solid tumors formed by K14E7 Fancd2^−/−^ clonal cells, and both LTBMC-derived IL-3 dependent and stromal cell lines from each genotype for the nuclear and/or cytoplasmic location of E7, p53, and Rb. Rb and p53 have been detected in nucleus and cytoplasm of many cell lines [68–69]. In stromal and IL-3 dependent cell lines from both K14E7 Fancd2^−/−^ and K14E7 Fancd2^+/+^ LTBMCs, but not Fancd2^−/−^ or (129/Sv X FVB F1) cell lines, there was detectable nuclear E7, as well as both p53 and Rb (Figure 7A–7F). These results establish that while fresh whole marrow from K14E7 Fancd2^−/−^ mice was negative for K14 or E7 protein, both K14 and the K14 promoter linked E7 protein were detected in hematopoietic and stromal cell lines derived from LTBMCs. Furthermore, K14 and E7 positive cell lines were derived from both K14E7 Fancd2^+/+^ and K14E7 Fancd2^−/−^ mouse LTBMCs, while only IL-3 suspension culture grown cell lines from the latter became FI and induced tumors *in vivo*.

### E6/E7 plasmid transfection of Fancd2^−/−^ IL-3 dependent cell lines increases cloning efficiency and malignant transformation

The above results establish that the E7 oncogene of HPV expressed in K14E7 Fancd2^−/−^ marrow LTBMCs generated FI cell lines that produced malignant plasma cell tumors. The evolution of malignant cell lines in LTBMCs involved the presence of both mesenchymal stem cells (stromal cells of the hematopoietic microenvironment), as well as hematopoietic progenitor cells. We next tested whether the E6 and E7 oncogenes of HPV could induce malignant transformation of hematopoietic cells independent of the marrow microenvironment. We utilized a plasmid containing both the E6 and E7 oncogenes of HPV and transfected Fancd2^−/−^ and control IL-3 dependent hematopoietic progenitor cells *in vitro* as described in the Methods. Stromal and IL-3 dependent hematopoietic cell lines from each genotype culture were transfected with this plasmid containing both E6 and E7. Single cell derived clonal lines were selected for G418 resistance according to the methods. No difference in morphology or growth was detected with any transfected stromal cell lines (Table 6). Transfected compared to control IL-3 dependent transfected single cell derived cell lines were expanded from single cells in multiwell plates in medium containing IL-3, and then were again expanded in medium with no IL3.

**Table 6:** E7 plasmid transfection of Fand2^−/−^IL-3 dependent cell line increases *in vitro* cloning efficiency

Cell Line	Single Cell Cloning Efficiency	Malignant Transformation of Expanded Single Cells
Stromal Cell Lines:		
K14E7 Fancd2^−/−^	52/400	0/400
K14E7 Fancd2^+/+^	48/400	0/400
Fancd2^−/−^	44/400	0/400
(129 X FVB) F1	48/400	0/400
IL-3 Dependent Subclonal Cell Lines (Plasmid Transfected):	(Control-Non-Transfected)	
Fancd2^−/−^(PLXSN16E6E7)	**164/400 (8/400)	**8/164 (0/8)
K14E7 Fancd2^+/+^	8/400 (6/400)	0/8 (0/6)
(129 X FVB) F1	6/400 (11/400)	0/6 (0/11)

Adherent stromal and IL-3 dependent cell lines were transfected with PLXSN16E6E7 plasmid, as described in the methods. Then, each of 400 single cells was plated in multi-well plates (in Il-3 containing medium). The number of cells containing a proliferating cell line was scored 7 days later. IL-3 dependent cell lines were then expanded in medium without IL-3. IL-3 independent (factor independent–FI) cell lines were those continuing to divide in the absence of IL-3.

** *p* <. 001 compared to non-transfected Fancd2^−/−^ cell line.

In contrast to all other negative results, E6/E7 plasmid transfected IL-3 dependent Fancd2^−/−^ cells showed an increase in cloning efficiency and IL-3 independent (factor-independent FI) cell lines were derived, which formed tumors *in vivo*. Tumor forming lines were detected with 8 of 164 single cell derived clonal lines (Table 6). None of 8 control non-transfected and expanded Fancd2^−/−^ single cell derived cell lines and no control or E6/E7 transfected Fancd2^+/+^ IL-3 dependent cell lines became IL-3 independent, and none formed tumors *in vivo* (Table 6). Therefore, Fancd2^−/−^ hematopoietic cells could be transformed by E6/E7 of HPV in the absence of stromal cells of the hematopoietic microenvironment (Table 6).

## DISCUSSION

The present studies demonstrate that the squamous cell lineage specific and restricted expression of E7 by the cytokeratin 14 promoter in K14E7 Fancd2^−/−^ mice was abrogated by growth of bone marrow in LTBMCs, LTBMC derived stromal and hematopoietic cells from K14E7 Fancd2^−/−^ mice expressed both K14 and E7. Furthermore, K14E7 Fancd2^−/−^ LTBMC derived IL-3 dependent cell lines, as well as freshly removed marrow hematopoietic progenitors were radiosensitive compared to those from Fancd2^−/−^mice. The relative radiosensitivity of K14E7 expressing hematopoietic cells was comparable to that reported for other oncogene [71], FA pathway [9, 73], or other double strand break repair gene altered cell lines including those from ATM mice [72].

Unexpectedly, K14E7 Fancd2^−/−^ LTBMC derived hematopoietic cells subcultured in IL-3 produced clonal malignant plasmacytoma cell tumors. K14E7 Fancd2^−/−^ mice are known to develop squamous cell tumors of the oral cavity and the female cervix [26], but in this model, either oral chemical carcinogen or estrogen pellet placement respectively was also required for carcinogenesis. In the present studies, untreated K14E7 Fancd2^−/−^ marrow in LTBMC and subcultured nonadherent cells grown in the presence of IL-3 spontaneously transformed to malignant plasmacytoma cell lines. We confirmed that K14E7 Fancd2^−/−^ mice treated with oral 4-NQO developed squamous cell tumors of the oral cavity and esophagus, but we saw no detectable effect of oral 4-NQO treatment of these tumor bearing mice on malignant transformation of their explanted bone marrow in LTBMCs.

The reduction in day 7 and day 14 colony forming cells in 4-NQO treated mouse, as well as in control mouse, K14 Fancd2^−/−^ LTBMCs was probably related to the reduction in stem cell numbers that was associated with the Fancd2^−/−^ genotype, prior chemical treatment and/or the malignant transformation of oral cavity that was in progress. In contrast, the effect of K14E7 expression in K14E7 Fancd2^+/+^ marrow cultures may have caused an increased cell production response, perhaps reflecting the effect of an intact Fancd2 gene with associated intact DNA repair. It is possible that the hydrocortisone added to LTBMC medium [28] and/or IL-3 added weekly to the subculture medium may have facilitated activation of the K14 promoter linked E7 expression in hematopoietic cells from K14E7 Fancd2^−/−^ and K14E7 Fand2^+/+^ mice; however, the Fancd2^−/−^ genotype was clearly required for malignant transformation of hematopoietic cells to plasmacytomas.

There was clearly an hematopoietic lineage specific biologic effect of adding the K14E7 genotype to Fancd2^−/−^ marrow cells when maintained in LTBMCs. Freshly removed organs from K14E7 Fancd2^−/−^ mice revealed E7 expression only in those tissues where cytokeratin 14 was naturally expressed, including: oral cavity, esophagus, skin, and cervix. There was no detectable K14 or E7 expression in kidney, liver, bone marrow, or spleen, even when these organs were explanted and grown in IL-3 containing medium for over 4 weeks. While LTBMC derived K14E7 Fancd2^−/−^ and K14E7 Fancd2+/+ cell lines showed expression of CK14 and its linked E7 oncogene protein in both marrow stromal and hematopoietic cells, only hematopoietic cell lines from the former generated malignant plasmacytoma cell lines *in vitro*.

The pattern of secondary growth of K14E7 Fancd2^−/−^ mouse nonadherent cells subcultured in IL-3 supplemented cultures was not detected with marrow from any of the other genotypes studied in the current experiments nor in prior publications [9–10]. The novel secondary culture growth pattern was also not observed with LTBMCs from over 28 other mouse strains [28]; nor has it been observed in leukemogenic retroviral or chemical carcinogen treated mouse LTBMCs [30–37].

The present K14E7 Fancd2^−/−^ malignant plasmacytomas derived from single cells expressed both kappa and lambda light chains, a phenomenon known to occur in human multiple myelomas [52]. Thus, the present results establish that expression of both light chains does not indicate a polyclonal tumor. Tumor cell lines, as well as IL-3 dependent cell lines derived of K14E7 Fancd2^−/−^ long-term cultures showed E7 binding to both nuclear p53 and Rb. While location of the E7 oncogene protein along with p53 and Rb was detected in the nucleus of K14E7 Fancd2^−/−^ LTBMC derived plasmacytoma tumor cell lines, we cannot conclude that these conditions were sufficient for malignant transformation, since non-tumorigenic stromal cell lines from K14E7 Fancd2^+/+^ mouse marrow cultures also showed nuclear location of these proteins. Direct transfection of IL-3 dependent, non-malignant cell lines derived from Fancd2^−/−^ long-term cultures with a plasmid containing both E6/E7, transformed 8 of 164 single cells to factor independence and the malignant phenotype. Increased growth and migration capacity, but not malignant transformation has been detected in KRAS transfected cord blood cells [70]. Whether hematopoietic growth factors other than IL-3 also stimulate malignant transformation of subcultured K14E7 Fancd2^−/−^ marrow cells from LTBMCs is not known. The clonal nature of K14E7 Fancd2^−/−^ cells with bilineage differentiation capacity was detected in 10 separate single cell derived subclonal lines. No bilineage clonal IL-3 dependent cell lines have been detected in LTBMCs from other mouse strains or with K14E7 Fancd2^−/−^ clonal cell lines grown in G-CSF, GM-CSF, IL-6, or IL-11 (not shown). Malignant plasmacytoma cell lines in our studies were growth factor independent. Recent data indicates that at least 12 different genes in the JAK/STAT and MAP Kinase pathways can induce transposon mutagenesis of IL-3 factor dependent cell lines to factor independence [66]. Whether the E7 oncogene in the absence of the Fancd2 protein induced these or other genes in our LTBMC derived cell lines is not known. Whether the E7 or E6 oncogene alone transforms IL-3 dependent Fancd2^−/−^ cell lines to malignant FI cell lines is not known since the plasmid used contained both oncogenes. Further studies will be required to determine if E7 (or E6) alone is sufficient for malignant transformation of Fancd2^−/−^ hematopoietic cells in the absence of stromal cells found in LTBMCs.

Complex effects of the HPV E6 and E7 oncogene in cell killing have been described [53–64]. A role for HPV E7 interaction with p53 has been shown in malignant transformation in that degradation of p53 was required for malignant transformation by HPV E6/E7 [65]. Whether degradation of p53 occurred in the malignant plasmacytoma cell lines derived from K14E7 Fancd2^−/−^ long-term marrow cultures is not known.

The present studies expand the possible cell target of HPV E7 to include the bone marrow in patients with FA. Replication of HPV has recently been shown to require DNA repair by homologous recombination (HR) and involving RAD51 and BRCA1 [23]. The HR pathway is known to be downregulated in FA cells, which are also sensitive to the effects of TGF-β [25]. There is published evidence that HPV alters hematopoietic cells. Plasma cell dyscrasias have been detected in patients with HPV infection [38], and patients with multiple myeloma have been shown to be HPV positive [39–40]. There is also evidence, which links HPV expression with malignant transformation of B-cell progenitors in the marrow, by co-transfection of HPV E7 or other oncogenes [41]. Human bone marrow cell lines expressing HPV16 E6 and E7 showed *in vitro* support capacity for hematopoiesis [43]. HPV16 E6/E7 immortalized human marrow stromal cell lines that support hematopoiesis showed integration of the E6/E7 vector into the cellular genome [43].

Fresh human bone marrow derived mesenchymal stem cells have been reported to become radiosensitive after HPV-16 E6/E7 transfection [44], and showed reduced DNA double strand break repair capacity and increased apoptosis. HPV mRNA expression has been detected after transfection of many other cell phenotypes including: mesenchymal stem cells [45], prostate cancer cell lines [46], keratinocytes [47], and dendritic cells [48]. Bone marrow transplantation has resulted in the transmission, from marrow of P16/human HPV to graft recipients resulting in oral squamous cell carcinoma [13]. While prior studies have shown that the E7 gene product activates multiple cell signaling pathways [24], malignant transformation of B-cell progenitor cells *in vitro* has not been reported. Evolution of B-cell and plasmacytoma-inducing tumors from adherent cells in culture has been reported with Epstein-Barr virus [49], and B-cell progenitors are known to reside in the adherent layer of long-term marrow cultures and can proliferate over time [50]. The Friend Spleen Focus Forming Virus (SFFV) expands hematopoietic islands in LTBMCs [51]; however, we detected no evidence of stem cell expansion in the present studies with HPV E7. The mechanism by which HPV E7 induces radiation sensitivity of hematopoietic cells and malignancy in K14E7 Fancd2^−/−^ mouse marrow cultures is not known. Further studies will be required to determine whether the condition(s) of LTBMC and/or IL-3 suspension culture are required for malignant transformation with HPV-E7 transfected freshly removed marrow stem cells from K14E7 Fancd2^−/−^ mice. The present results on the biology of K14E7 Fancd2^−/−^ marrow in LTBMCs provide a new system in which to study effects of HPV on: 1) the mechanism of HPV E6/7 genes in tumorigenesis in an *in vitro* organ culture system; 2) the mechanism of the K14 promoter control of expression of E7 in mesenchymal and hematopoietic cells in culture; and 3) the effect of an absent Fancd2 protein on expansion of a subpopulation of primitive adult bone marrow cells to both hematopoietic and mesenchymal stromal cell phenotypes.

Fanconi Anemia patients may be at increased risk of cell transformation by HPV, in part attributable to defective DNA repair [6, 15, 53]. Fancd2^−/−^ mice of both the 129/Sv [10] and C57BL/6J [9] background strains demonstrate significant radiosensitivity. Clinical radiotherapy patients with HPV positive head and neck cancers have been reported to have tumor control by lower radiation doses [17]. While both Fancd2^−/−^ and K14E7 Fancd2^−/−^ marrow stromal lines in our studies were radiosensitive, only Fancd2^−/−^hematopoietic cell lines and fresh marrow colony forming progenitor cells were radioresistant. These data indicate that the Fancd2 protein has phenotype-specific effects on the ionizing irradiation response. The present data demonstrate that the E7 oncogene of HPV transforms Fancd2^−/−^ hematopoietic cells in LTBMCs to malignant plasma cell tumors, and extend the spectrum of potential effects of HPV to the bone marrow of genetically susceptible patients with DNA repair defects including those with Fanconi Anemia.

## MATERIALS AND METHODS

### Mice

Fancd2^−/−^, Fancd2^+/−^, and Fancd2^+/+^ (129/Sv background [1]) and K14E7 mice (FVB background [26]) were generously provided by Dr. Paul Lambert (University of Wisconsin, Madison, WI). Mice were bred according to published methods to produce K14E7Fancd2^−/−^ mice [26] as published.

### 4-nitro-quinolone-oxide (4-NQO) treatment

In subgroups of mice, the addition of 4-nitro-quinolone oxide (4-NQO) (Sigma Chemical Company, St. Louis, MO) to drinking water was carried out as published [14]. Briefly, 4-NQO (10 μg/ml) was added to the drinking water weekly for 8 weeks. The mice were followed for development of tumors or sacrificed after 15 weeks then bone marrow was isolated for establishment of Long Term Bone Marrow Cultures [28]. The original studies, Park, et al. [14] showed that 3 conditions were required for oral cavity cancer development in K14E7 Fancd2^−/−^ mice: 1) the absence of Fancd2; 2) the E7 oncogene; and 3) exposure to the 4-NQO carcinogen.

### Cell lines

Bone marrow stromal cell lines and Interleukin-3 dependent cell lines from Fancd2^−/−^ (129/Sv) [10], Fancd2^−/−^ (C57BL/6) [9], and control mouse strains have been reported. The mouse HPV-induced cytokeratin 14 positive squamous cell tumor cell (CaSki) line was obtained from American Type Culture Collection (Manassas, VA). A clonal 3LL mouse (C57BL/6) Lewis Lung Carcinoma cell line [67] and a C57BL/6J control mouse spontaneous colon cancer were explanted to culture, and cell lines were established by us.

### Long-term bone marrow cultures

Long-term bone marrow cultures (LTBMC) were established from the femur and tibia marrow of (129/Sv background) Fancd2^+/+^, Fancd2^+/−^, and (FVB/n background) K14E7Fancd2^−/−^ mice according to published methods [9]. Briefly, the contents of a femur and tibia were flushed into McCoy's 5A medium (Gibco, Gaithersburg, MD) supplemented with 25% horse serum (Cambrex, Rockland, ME), and 10^−5^ M hydrocortisone sodium hemisuccinate. Cultures were incubated at 33°C in 7% CO_2_. After 4 weeks, the horse serum was replaced with 25% FBS (Gibco, Gaithersburg, MD) [9]. The cultures were observed weekly for hematopoietic cell production and cobblestone island formation. Cobblestone islands of greater than or equal to 50 cells were scored weekly in each flask [9]. A two-sided two-sample *t-test* was used to compare the number of cobblestone islands between each genotype cultures each week. *P*-values less than 0.05 were regarded as significant.

### Hematopoietic cell colony-forming assays

Aliquot of 5 × 10^4^ fresh bone marrow cells or nonadherent cells removed at week 4 from each genotype long-term marrow culture were plated in triplicate in semi-solid medium consisting of methylcellulose in IMDM, using a GelCount colony counter (Oxford Optronix, Oxford, UK) and maintained in a high humidity incubator according to published methods and using growth factor supplements as described [9]. Briefly, cells from fresh marrow or primary or subclonal IL-3-dependent cell lines were plated in 0.8% methylcellulose supplemented with 10% Iscove's Modified Dulbecco's Medium (IMDM), 30% fetal bovine serum (FBS), 1% bovine serum albumin, 2 ng/mL IL-3 (Stemcell Technologies, Vancouver, Canada) at variable cell densities. At day 14, individual colonies were harvested and each cultured in a well of a 96 well plate in 0.2 mL of IMDM supplemented with 30% FBS and 1 ng/mL of Interleukin-3 (IL-3). Colonies of greater than 50 cells were scored on day 7. Data were analyzed with single-hit multitarget models according to published methods [9].

### Establishment of IL-3-dependent hematopoietic progenitor cell lines and clonal cell sub-lines

Non-adherent cells were harvested from each genotype mouse LTBMC at week 4 and cultured in six-well tissue culture plates in Iscove's Modified Eagles Medium (IMDM) supplemented with 20% fetal calf serum (FBS) and 1.0 ng/mL recombinant mouse IL-3 (Peprotech, Rocky Hill, NJ). The cell lines were passaged weekly for 12–14 weeks to establish primary IL-3-dependent cell lines using published methods [9].

Clonal cell sub-lines were established from each of the genotype parent cell lines by expansion of single cell derived colonies. Cells were then replated in methylcellulose, colonies selected at 14 days, and cultured as above, to establish subclonal lines [9]. Confirmation of genotype after repeated subcloning was carried out for each cell line.

### Establishment of bone marrow stromal cell Lines and clonal cell sub-lines

Adherent cell layers from one 4-week old LTBMC from each genotype mice were trypsinized and expanded by passage into Dulbecco's Modified Eagle's Medium (DMEM) + 10% FBS to establish bone marrow stromal cell lines according to published methods [9]. Cells were passaged for 10 weeks to establish cell lines. Cultures were incubated at 37^°^C in 5% CO_2_.

### Flow cytometry analysis for cell surface phenotype

Flow Analysis for CD3c, CD5, CD8, B220, GR1–Ter119, CD41, CD45, CD48, SCA-1, C-KIT, CD150, vimentin, adiponectin, collagen III, collagen IV, osteopontin, and osteocalcin (antibodies were obtained from Abcam Inc., Cambridge, MA or Santa Cruz Biotechnology, Inc., Santa Cruz, CA) were carried out according to the methods section sorting 10,000 cells in triplicate experiments. Briefly, bone marrow stromal cells and IL-3 dependent hematopoietic cells from K14E7 Fancd2^−/−^ and SV129 Fancd2^−/−^ cells were prepared into single cell suspension and incubated with each FITC or PE conjugated antibody listed above at concentrations recommended by the manufacturer for one hour on ice. The cells were washed three times with phosphate buffered saline (PBS) and analyzed for positive cells by flow cytometry.

### Western blot for proteins

Cell lines were cultured in DMEM medium (Lonza Cat. #: 12-604F) with 10% Fetal Bovine serum (Gemini Cat.#: 100–500), 1% L-Glutamine (Lonza Cat. #: 17-605E) and 1% Antibiotics Antimycotic Solution (Corning Cat. #: 30-004-Cl). Total cellular protein was extracted using protein extraction buffer (IP Lysis Buffer, Thermo Scientific prod #: 87787), containing protease inhibitor and phosphatase inhibitor cocktails (Thermo Scientific prod #: 78442). Mouse tongue and skin tissue were freshly taken and chopped to fine pieces and homogenized in protein extraction buffer (same as cell protein extraction buffer). Protein concentration was determined using the Bio-Rad protein assay system (Bio-Rad Laboratories, Cat. #: 500–0006). The proteins (15 μg per lane) were separated on denaturing polyacrylamide gels (Bio-Rad Laboratories, Mini-Protean TGX Gels Cat. #: 456–1083) and then transferred to PVDF membranes (Bio-Rad Laboratories, Immun-Blot PVDF Cat. #: 162–0177) by electrophoresis. Blots were blocked with 5% Fat-free Dry Milk in TBST for 1 h and then incubated overnight with primary antibodies (see antibody table). The membranes were washed with TBST and processed with corresponding horseradish peroxidase-conjugated secondary antibodies (see antibody table). The proteins were exposed to x-ray film (5 to 30 seconds) using ECL detection reagent (Thermo Scientific SuperSignal West Dura Extended Duration Substrate Prod #: 34075). To ensure equal protein loading, the same blot was subsequently developed for GAPDH expression.

K6, K16, and K17 are found in oral mucosa and stratified epidermis of skin [27]. K17 has been reported to be found in normal skin and in malignant squamous cell lesions [27]. CK13 is expressed in normal epithelium, and is reduced after malignant transformation [27]. K10 is an early differentiation marker, found in the suprabasal epidermal layer in uninvolved skin with reduced expression in malignant lesion areas [27].

### Immunohistochemistry

We used control anti-CK-13 antibody (ab92551) and anti-CK14 (ab7800) antibody for staining squamous cell specific Cytokeratins. For Kappa and Lambda staining, goat anti-Lambda light chain (Bethyl, Inc. A90-121A) and goat anti-kappa light chain (Bethyl, Inc. A90-119A), K14E7 Fanconi d2^−/−^ cells were seeded and grown in chamber slides and fixed with 100% methanol (5 min), permeabilized with 0.2% Triton X-100 for 10 minutes and blocked with 1% BSA/22.5 mg/ml glycine in 0.1% PBS-Tween for 30 minutes. The cells were then incubated overnight at +4^°^C with the antibody (ab92551) or (ab7800) at a 1/100 dilution, then incubated with secondary antibody (Dnk anti-rabbit alexa flour 488 or Dnk anti-goat alexa flour 488) for 1 hour at a 1/500 dilution (shown in green). Nuclear DNA was labeled with DAPI (shown in blue). Images were obtained using a Nikon Eclipse E800 microscope.

### Immunofluorescence

Tumors were fixed using 10% paraformaldehyde, embedded in paraffin, sectioned, and deparaffinized in xylene for 10 min twice and rehydrated in 100%, 95%, and 70% ethanol for 5 min. Antigens were unmasked by boiling for 12 min in 1 mM EDTA. Sections were blocked with 3% H2O2 at room temperature for 30 min, washed in PBS for 5 min × 3, incubating in 10% BSA at room temperature for 15 min. The sections were incubated with the primary antibody at 4°C overnight, and washed in PBS for 5 min × 3. Secondary antibody was added for 1 hr at room temperature and washed in PBS for 5 min × 3. The sections were incubated in Elite ABC Kit (Vector #PK-6100) at room temperature for 30 min, washed in PBS for 5 min × 3, incubated in DAB kit (Vector #SK-4100) at room temperature for 1 min, and washed in running water for 5 min. Hematoxylin was added for one followed by Methylene Blue Reagent Solution for 1 min. The sections were dehydrated in 70%, 95%, and 100% ethanol followed by xylene for 5 min. the slides were mounted and analyzed.

### Clonogenic irradiation survival curves for K14E7 Fancd2^−/−^ mouse IL-3-dependent cell lines or freshly removed whole bone marrow

IL-3-dependent non-adherent cells or whole bone marrow from each genotype mice were irradiated in suspension culture to doses between 0 and 8 Gy as described above. Cells were plated in triplicate in methylcellulose medium containing recombinant mouse stem cell factor (SCF), IL-3, IL-6, and recombinant human erythropoietin (EPO) (Stem Cell Technologies, Vancouver, BC). CFU-GM were scored on day 7–9 for the IL-3-dependent cell lines and CFU-GM, BFU-E, and CFU-GEMM were scored between days 10 and 13 for the whole bone marrow. In some experiments, colonies growing in plates containing cells irradiated to 6 Gy or 8 Gy were removed and expanded. Data were analyzed with single-hit multitarget models according to published methods [9].

In other experiments with fresh marrow, cells were plated in semisolid medium with Fetal Bovine Serum (FBS), 10% Bovine Serum Albumin (BSA), recombinant mouse IL-3, also containing L-glutamine, 3 U/mL erythropoietin, and 2-mercaptoethanol. Fresh marrow colonies were scored at day 7 and 14 and subdivided as colony forming unit granulocyte macrophage (CFU-GM), Burst Forming Unit Erythroid (BFUe), and (CFU-GEMM) granulocyte-erythroid-megakaryocyte-macrophage. Colony forming unit granulocyte-macrophage (CFU-GM) burst forming erythroid (BFUe) and (CFU-GEMM) of 50 cells or greater were counted on both days 7 and 14 after plating. A two-sided, two-sample *t-test* was used as described above [9].

**Table:** List of premium antibodies used for western immunoblotting studies

Antibodies	Dilution	Source	Catalogue Numbers
Mouse anti CK-5	1:1000	Abcam	ab128190
Rabbit anti CK-6	1:1000	Abcam	ab52869
Rabbit anti CK-10	1:500	Abcam	ab76318
Rabbit anti CK-13	1:1000	Abcam	ab92551
Mouse anti CK-14	1:1000	Abcam	ab7800
Rabbit anti CK-16	1:1000	Abcam	ab182791
Rabbit anti CK-17	1:500	Abcam	ab109725
Mouse anti GAPDH	1:2000	Millipore	MAB374
Mouse anti HPV E7	1:500	Santa Cruz	Sc-6981

**Table:** List of secondary antibodies used for western immunoblotting studies

Secondary Antibodies	Dilution	Source	Catalogue Number
Anti-Rabbit IgG, HRP Conjugate	1:15000	Promega	W401B
Anti-Mouse IgG, HRP Conjugate	1:15000	Promega	W402B

### Clonogenic irradiation survival curves for K14E7Fancd2^−/−^ mouse bone marrow stromal cell lines

Bone marrow stromal cell lines generated from the trypsinized and repassaged LTBMC adherent layer at week 4 [9] from each genotype mouse strain were subcloned in single cell counting wells of micro-well plates as described [9]. Clonal cell lines were expanded. Cells were then trypsinized and irradiated in suspension culture to doses between 0 and 8 Gy at 70 cGy/min using a Shepherd Mark 1 ^137^Cs ƴ-ray source (J.L. Shepherd, San Fernando, CA, USA). Cells were plated in quadruplicate in Linbro plates (Fisher Scientific, Pittsburgh, PA, USA) and incubated at 37^°^C and 5% CO_2_ for 9–11 days, stained with crystal violet, and colonies of > 50 cells were counted.

### E6/E7 plasmid transfection of stromal and IL-3 dependent hematopoietic progenitor cell lines

IL-3 dependent nonadherent cell lines derived from each genotype LTBMC or clonal stromal cell lines were transfected with the PLXSN16E6E7 plasmid containing both E6 and E7; PLXSN16E7 (Addgene, Inc., Cambridge, MA) according to the manufacturer's directions. Selection of G418 resistant clones was carried out with transfected and control IL-3 dependent hematopoietic cell lines including cell lines: 32D cl3 [29], as well as cell lines derived from Fancd2^+/+^ (129/Sv) [10], Fancd2^−/−^ (129/Sv) [10], and cell lines derived from control 129/Sv, FVB/n, and (129/Sv X FVB/n)-F1 long-term marrow cultures.

### Cytokeratin and E7 HPV oncogene expression in cell lines and fresh tissues

Protein was extracted from fresh tissues or cell lines from each mouse strain including: 129/Sv Fancd2^+/+^, 129/Sv Fancd2^−/−^, K14E7 Fancd2^+/+^, and K14E7 Fancd2^−/−^ mice. We first studied bone marrow stromal cell lines and IL-3 dependent cell lines as well as freshly removed fresh tissue from K14E7 Fancd2^+/+^ and K14E7 Fancd2^−/−^ mice. Protein was extracted using protein extraction buffer (IP Lysis Buffer, Thermo Scientific prod #: 87787), containing protease inhibitor and phosphatase inhibitor cocktails (Thermo Scientific prod #: 78442). Protein concentration was determined using the Bio-Rad protein assay system (Bio-Rad Laboratories, Cat. #: 500-0006). The proteins (15 μg per lane) were separated on denaturing polyacrylamide gels (Bio-Rad Laboratories, Mini-Protean TGX Gels Cat. #: 456-1083), and then transferred to PVDF membranes (Bio-Rad Laboratories, Immun-Blot PVDF Cat. #: 162-0177) by electrophoresis. Blots were blocked with 5% Fat-free Dry Milk in TBST for 1 h and then incubated overnight with primary antibodies to mouse anti-CK14 (ab7800 Abcam, Cambridge, MA), or mouse anti-HPV E7 protein (SC6981, Santa Cruz Biotechnology, Santa Cruz, CA). The membranes were then washed with TBST and processed with a corresponding horseradish peroxidase-conjugated secondary antibodies (HRP anti mouse IgG, Promega, Madison, WI). The blotted proteins were then exposed to x-ray film (5 to 30 seconds) using ECL detection reagent (Thermo Scientific SuperSignal West Dura Extended Duration Substrate Prod. #: 34075). To ensure equal protein loading, each blot was also subsequently developed for level of GAPDH (glyceraldehyde-3-phosphate-dehydrogenase) expression.

### Tumor formation assays

To demonstrate that K14E7 Fancd2^−/−^ clonal cell lines were tumorigenic, 1 × 10^6^ cells from K14E7 Fancd2^−/−^ clone 1 or K14E7 Fancd2^+/+^ clone 5 cell lines were injected subcutaneously in the flank of each of 5 (FVB X 129/Sv) F1 mice. Other groups of 5 mice received I.V. injection of the same cell numbers. Mice were followed for the development of tumors at which time they were sacrificed, tumors removed, fixed in 10% paraformaldehyde, sectioned, and then sections were stained with hematoxylin and eosin or antibodies to kappa and lambda light chains (A90-121A or A90-119A, Bethyl Laboratories, Montgomery, TX).

### Detection of E7 oncogene protein with nuclear p53 and Rb

The primary antibodies used for co-localization studies included: a rabbit polyclonal antibody to p53 (Santa Cruz Biotechnology, Santa Cruz, CA, sc-6243); a rabbit polyclonal antibody to Rb (Santa Cruz Biotechnology, Santa Cruz, CA, sc-50); and a mouse monoclonal antibody to E7 (Santa Cruz Biotechnology, Santa Cruz, CA, sc-65711). Secondary antibodies used included: donkey anti-mouse 488 and donkey anti-rabbit 568 (obtained from Invitrogen, Waltham, MA). DAPI was used as a nuclear fluorescent stain that binds strongly to A-T rich regions in DNA. Confocal images were obtained with an Olympus Fluoview 100 confocal microscope and companion software FV10-ASW1.6. Images were acquired at a resolution of 1024 × 1024 pixels.

### Statistical methods

The *in vitro* radiation survival curves were analyzed with the single-hit multi-target model, and were compared using D_0_ (final slope representing multiple-event killing) and ñ (extrapolation number measuring width of the shoulder on the radiation survival curve) [9]. Results for D_0_ and ñ were presented as the mean ± standard error (SEM) from multiple measurements and compared with the two-sided two-sample *t-test*.

For the LTBMC data, weekly cobblestone island numbers; non-adherent cell numbers (×10^6^) per flask; percent confluence of adherent cells; day 7 colony forming progenitor cells; and day 14 colony forming cells were counted as described [9] and compared at each week between any two of the four groups (i.e., K14E7 Fancd2^+/+^, K14E7 Fancd2^−/−^, control (129/Sv × FVB) F_1_ and Fancd2 ^−/−^ (129/Sv). Data were summarized as mean ± standard deviation, and *p*-values were calculated with the two-sided two-sample *t-test*.

For Western blots data were analyzed by densitometry and summarized with mean ± standard deviation in each group. *P*-values for the comparison between any two groups were calculated with the two-sided two-sample *t-test*.

For the other continuous endpoints, comparisons were also performed using a *t-test* if they were normally distributed, or with Wilcoxon rank sum test otherwise. In all these tests, a *p-value* of less than 0.05 was regarded as significant. As an exploratory analysis, we did not adjust *p*-values for multiple comparisons.

## SUPPLEMENTARY MATERIALS TABLES












