# Supplementary material for: Evolution of malignant plasmacytoma cell lines from K14E7 Fancd2−/− mouse long-term bone marrow cultures
Source: Oncotarget. 2016 Sep 15;7(42):68449–72. doi: 10.18632/oncotarget.12036 (PMC5356567; doi:10.18632/oncotarget.12036)
Supplement: Supplementary file 1 [file oncotarget-07-68449-s001.pdf]

# Evolution of malignant plasmacytoma cell lines from K14E7 Fancd2$^{-/-}$ mouse long-term bone marrow cultures

**Supplementary Materials**

**Supplementary Table S1: Analysis of percent confluence in LTBMCs from K14E7 Fancd2$^{-/-}$ mice**. See Supplementary_Table_S1

**Supplementary Table S2: Analysis of cobblestone islands in K14E7 Fancd2$^{-/-}$ LTBMCs.** See Supplementary_Table_S2

**Supplementary Table S3: Analysis of non-adherent cells per flask from LTBMCs of a K14E7 Fancd2$^{-/-}$ mice (× 1000000).** See Supplementary_Table_S3

**Supplementary Table S4: Analysis of day 7 colony forming progenitor cells from K14E7 Fancd2$^{-/-}$ LTBMCs.** See Supplementary_Table_S4

**Supplementary Table S5: Analysis of day 14 colony forming cells from LTBMCs from K14E7 Fancd2$^{-/-}$ mice.** See Supplementary_Table_S5

**Supplementary Table S6: Summary of K14 and E7 expression by Western blot and histochemical staining on tumorigenic K14E7 Fancd2$^{-/-}$ marrow derived hematopoietic stromal cell lines**

| Cell Line | E7 Western | E7 Staining | CK14 Western | CK14 Staining |
|---|---|---|---|---|
| 1. B6 FancD2+/+ (adherent) | Negative | Negative | Negative | Negative |
| 2. B6 FancD2−/− (adherent) | Negative | Negative | Negative | Negative |
| 3. K14/E7 FancD2+/+ (adherent) | Positive | Positive | Positive | Positive |
| 4. K14/E7 Fancd2−/− (adherent) | Positive | Positive | Positive | Positive |
| 5. B6 Fancd2+/+ (IL-3 dependent) | Negative | Negative | Negative | Negative |
| 6. B6 Fancd2 Fancd2−/− (IL-3 dependent) | Negative | Negative | Negative | Negative |
| 7. K14/E7 Fancd2+/+ (IL-3 dependent) | Positive | Positive | Positive | Positive |
| 8. K14/E7 Fancd2−/− (IL-3 dependent) | Positive | Positive | Positive | Positive |
