# Supplementary material for: Evolution of malignant plasmacytoma cell lines from K14E7 Fancd2−/− mouse long-term bone marrow cultures
Source: Oncotarget. 2016 Sep 15;7(42):68449–72. doi: 10.18632/oncotarget.12036 (PMC5356567; doi:10.18632/oncotarget.12036)
Supplement: Supplementary file 2 [file oncotarget-07-68449-s002.docx]

**Supplemental Table 1: Analysis of percent confluence in LTBMCs from K14E7 Fancd2^-/-^ mice.**

| group | **Week 1** | **Week 2** | **Week 3** | **Week 4** | **Week 5** | **Week 6** |
| --- | --- | --- | --- | --- | --- | --- |
| K14E7 Fancd2 -/- | 25.0±0.0 (n=2) | 77.5±3.5 (n=2) | 85.0±0.0 (n=2) | 90.0±0.0 (n=2) | 85.0±0.0 (n=2) | 87.5±3.5 (n=2) |
| K14E7 Fancd2+/+ | 35.0±0.0 (n=2) p1<0.0001 | 85.0±0.0 (n=2) p1=0.20 | 80.0±0.0 (n=2) p1<0.0001 | 85.0±0.0 (n=2) p1<0.0001 | 85.0±0.0 (n=2)  p1= 1.00 | 85.0±0.0 (n=2) p1=0.50 |
| FancD2-/- | 26.3±2.5 (n=4) p1=0.39 p2=0.0060 | 61.3±8.5 (n=4) p1=0.069 p2=0.012 | 70.0±4.1 (n=4) p1=0.0052 p2=0.016 | 77.5±2.9 (n=4) p1=0.0032 p2=0.014 | 82.5±5.0 (n=4) p1=0.39 p2=0.39 | 90.0±0.0 (n=4) p1=0.50 p2<0.0001 |
| Fancd2+/+ | 32.5±2.9 (n=4) p1=0.014 p2=0.18 p3=0.017 | 85.0±0.0 (n=4) p1=0.20  p2= 1.00 p3=0.012 | 78.8±2.5 (n=4) p1=0.015 p2=0.39 p3=0.011 | 80.0±0.0 (n=4) p1<0.0001 p2<0.0001 p3=0.18 | 75.0±0.0 (n=4) p1<0.0001 p2<0.0001 p3=0.058 | 85.0±0.0 (n=4) p1=0.50  p2=1.00 p3<0.0001 |
| group | **Week 7** | **Week 8** | **Week 9** | **Week 10** | **Week 11** | **Week 12** |
| K14E7 Fancd2 -/- | 90.0±0.0 (n=2) | 85.0±0.0 (n=2) | 87.5±3.5 (n=2) | 87.5±3.5 (n=2) | 90.0±0.0 (n=2) | 90.0±0.0 (n=2) |
| K14E7 Fancd2+/+ | 90.0±0.0 (n=2) p1=1.00 | 85.0±0.0 (n=2) p1=1.00 | 90.0±0.0 (n=2) p1=0.50 | 90.0±0.0 (n=2) p1=0.50 | 90.0±0.0 (n=2) p1=1.00 | 90.0±0.0 (n=2) p1=1.00 |
| Fancd2 -/- | 95.0±0.0 (n=4) p1<0.0001 p2<0.0001 | 95.0±0.0 (n=4) p1<0.0001 p2<0.0001 | 95.0±0.0 (n=4) p1=0.20 p2<0.0001 | 95.0±0.0 (n=4) p1=0.20 p2<0.0001 | 95.0±0.0 (n=4) p1<0.0001 p2<0.0001 | 95.0±0.0 (n=4) p1<0.0001 p2<0.0001 |
| Fancd2+/+ | 86.3±2.5 (n=4) p1=0.057 p2=0.057 p3=0.0060 | 86.3±2.5 (n=4) p1=0.39 p2=0.39 p3=0.0060 | 85.0±0.0 (n=4) p1=0.50 p2<0.0001 p3<0.0001 | 85.0±0.0 (n=4) p1=0.50 p2<0.0001 p3<0.0001 | 85.0±0.0 (n=4) p1<0.0001 p2<0.0001 p3<0.0001 | 87.5±2.9 (n=4) p1=0.18  p2=0.18  p3=0.014 |
| group | **Week 13** | **Week 14** | **Week 15** | **Week 16** | **Week 17** | **Week 18** |
| K14E7 Fancd2 -/- | 90.0±0.0 (n=2) | 90.0±0.0 (n=2) | 90.0±0.0 (n=2) | 90.0±0.0 (n=2) | 90.0±0.0 (n=2) | 90.0±0.0 (n=2) |
| K14E7 Fancd2+/+ | 90.0±0.0 (n=2) p1=1.00 | 90.0±0.0 (n=2) p1=1.00 | 90.0±0.0 (n=2) p1=1.00 | 90.0±0.0 (n=2) p1=1.00 | 90.0±0.0 (n=2) p1=1.00 | 90.0±0.0 (n=2) p1=1.00 |
| Fancd2 -/- | 95.0±0.0 (n=4) p1<0.0001 p2<0.0001 | 95.0±0.0 (n=4) p1<0.0001 p2<0.0001 | 95.0±0.0 (n=4) p1<0.0001 p2<0.0001 | 95.0±0.0 (n=4) p1<0.0001 p2<0.0001 | 95.0±0.0 (n=4) p1<0.0001 p2<0.0001 | 95.0±0.0 (n=4) p1<0.0001 p2<0.0001 |
| Fancd2+/+ | 88.3±2.9 (n=3) p1=0.42 p2=0.42 p3=0.057 | 88.3±2.9 (n=3) p1=0.42 p2=0.42 p3=0.057 | 88.3±2.9 (n=3) p1=0.42 p2=0.42 p3=0.057 | 90.0±0.0 (n=2) p1=1.00 p2=1.00 p3<0.0001 | 90.0±0.0 (n=2) p1=1.00 p2=1.00 p3<0.0001 | 90.0±0.0 (n=2) p1=1.00  p2=1.00 p3<0.0001 |
| group | **Week 19** | **Week 20** | **Week 21** |  |  |  |
| K14E7 Fancd2 -/- | No data | No data | No data |  |  |  |
| K14E7 Fancd2+/+ | No data | No data | No data |  |  |  |
| Fancd2 -/- | 95.0±0.0 (n=4) | 95.0±0.0 (n=4) | 95.0±0.0 (n=4) |  |  |  |
| Fancd2+/+ | 90.0±0.0 (n=2) p3<0.0001 | 90.0±0.0 (n=2) p3<0.0001 | 90.0±0.0 (n=2) p3<0.0001 |  |  |  |

Data are summarized with mean + standard deviation, and compared with the two-sided two-sample t-test, where P1 is the p-value for the comparison with K14E7 Fancd2^-/-^; P2 is the p-value for the comparison with K14E7 Fancd2^+/+^; and P3 is the p-value for the comparison with Fancd2^-/-^.
