# Supplementary material for: Evolution of malignant plasmacytoma cell lines from K14E7 Fancd2−/− mouse long-term bone marrow cultures
Source: Oncotarget. 2016 Sep 15;7(42):68449–72. doi: 10.18632/oncotarget.12036 (PMC5356567; doi:10.18632/oncotarget.12036)
Supplement: Supplementary file 3 [file oncotarget-07-68449-s003.docx]

**Supplemental Table 2: Analysis of cobblestone islands in K14E7 Fancd2^-/-^ LTBMCs**

| group | **Week 1** | **Week 2** | **Week 3** | **Week 4** | **Week 5** | **Week 6** |
| --- | --- | --- | --- | --- | --- | --- |
| K14E7 Fancd2 -/- | 77.0±1.4 (n=2) | 834.0±8.5 (n=2) | 1422.0±93.3 (n=2) | 1102.0±246.1 (n=2) | 1006.0±65.1 (n=2) | 1040.0±294.2 (n=2) |
| K14E7 Fancd2+/+ | 476.0±130.1 (n=2)  p1=0.14 | 1778.0±229.1 (n=2) p1=0.11 | 1926.0±31.1 (n=2)  p1=0.019 | 1640.0±73.5 (n=2)  p1=0.098 | 1282.0±138.6 (n=2)  p1=0.13 | 1492.0±231.9 (n=2)  p1=0.23 |
| Fancd2 -/- | 102.0±74.3 (n=4)  p1=0.55 p2=0.0092 | 832.5±516.5 (n=4) p1=1.00 p2=0.077 | 1058.0±606.5 (n=4)  p1=0.47 p2=0.13 | 519.0±307.6 (n=4)  p1=0.084 p2=0.0086 | 667.0±547.0 (n=4)  p1=0.46  p2=0.21 | 756.0±67.6 (n=4) p1=0.39 p2=0.0028 |
| Fancd2+/+ | 327.0±114.8 (n=4)  p1=0.022 p2=0.22 p3=0.017 | 2184.0±332.8 (n=4) p1=0.0039 p2=0.20 p3=0.0046 | 1914.5±233.9 (n=4)  p1=0.052 p2=0.95 p3=0.039 | 1501.0±613.1 (n=4)  p1=0.45 p2=0.78 p3=0.029 | 1558.0±108.4 (n=4) p1=0.0030 p2=0.052 p3=0.045 | 2002.0±346.5 (n=4)  p1=0.029  p2=0.14 p3=0.0046 |
| group | **Week 7** | **Week 8** | **Week 9** | **Week 10** | **Week 11** | **Week 12** |
| K14E7 Fancd2 -/- | 772.0±118.8 (n=2) | 348.0±62.2 (n=2) | 295.0±72.1 (n=2) | 153.0±66.5 (n=2) | 100.0±17.0 (n=2) | 82.0±93.3 (n=2) |
| K14E7 Fancd2+/+ | 1305.0±210.7 (n=2)  p1=0.089 | 1602.0±234.8 (n=2) p1=0.018 | 1392.0±79.2 (n=2) p1=0.0047 | 1044.0±509.1 (n=2)  p1=0.13 | 592.0±67.9 (n=2) p1=0.010 | 552.0±203.6 (n=2)  p1=0.097 |
| Fancd2 -/- | 885.0±565.9 (n=4)  p1=0.80 p2=0.39 | 460.0±171.7 (n=4) p1=0.44 p2=0.0022 | 288.0±129.1 (n=4)  p1=0.95 p2=0.0004 | 147.5±57.4 (n=4)  p1=0.92 p2=0.24 | 223.3±145.6 (n=3)  p1=0.33 p2=0.048 | 122.7±29.5 (n=3) p1=0.51  p2=0.20 |
| Fancd2+/+ | 2176.0±137.5 (n=4) p1=0.0003 p2=0.0032 p3=0.017 | 1562.0±759.2 (n=4) p1=0.10 p2=0.95 p3=0.059 | 1080.0±892.5 (n=4)  p1=0.31 p2=0.67 p3=0.17 | 772.0±692.7 (n=4)  p1=0.30 p2=0.66 p3=0.17 | 889.0±648.7 (n=4)  p1=0.093 p2=0.58 p3=0.15 | 931.0±685.1 (n=4)  p1=0.17  p2=0.51  p3=0.099 |
| group | **Week 13** | **Week 14** | **Week 15** | **Week 16** | **Week 17** | **Week 18** |
| K14E7 Fancd2 -/- | 66.0±36.8 (n=2) | 46.0±14.1 (n=2) | 90.0±8.5 (n=2) | 106.0±14.1 (n=2) | 122.0±2.8 (n=2) | 166.0±14.1 (n=2) |
| K14E7 Fancd2+/+ | 262.0±25.5 (n=2)  p1=0.025 | 214.0±36.8 (n=2) p1=0.026 | 328.0±39.6 (n=2)  p1=0.014 | 336.0±11.3 (n=2)  p1=0.0031 | 334.0±70.7 (n=2) p1=0.052 | 274.0±2.8  (n=2)  p1=0.0088 |
| Fancd2 -/- | 298.0±222.5 (n=3)  p1=0.26 p2=0.84 | 66.7±9.2 (n=3) p1=0.13 p2=0.0056 | 52.0±14.4 (n=3)  p1=0.047 p2=0.0013 | 70.0±2.8 (n=2) p1=0.072  p2=0.0010 | 32.0±17.0 (n=2)  p1=0.018 p2=0.028 | 109.0±29.7 (n=2) p1=0.13  p2=0.016 |
| Fancd2+/+ | 912.0±796.1 (n=3)  p1=0.25  p2=0.29 p3=0.27 | 277.3±290.8 (n=3) p1=0.36 p2=0.79 p3=0.34 | 402.7±545.9 (n=3)  p1=0.43 p2=0.87 p3=0.38 | 616.0±871.2 (n=2)  p1=0.56  p2=0.73 p3=0.54 | 144.0±203.6 (n=2)  p1=0.90 p2=0.34 p3=0.52 | 304.0±429.9 (n=2)  p1=0.73  p2=0.94  p3=0.59 |
| group | **Week 19** | **Week 20** | **Week 21** |  |  |  |
| K14E7 Fancd2 -/- | No data | No data | No data |  |  |  |
| K14E7 Fancd2+/+ | No data | No data | No data |  |  |  |
| Fancd2 -/- | 80.0±11.3 (n=2) | 24.0±5.7 (n=2) | 48.0±11.3 (n=2) |  |  |  |
| Fancd2+/+ | 250.0±353.6 (n=2)  p3=0.62 | 137.0±193.7 (n=2) p3=0.56 | 100.0±141.4 (n=2)  p3=0.66 |  |  |  |

Data are summarized with mean + standard deviation, and compared with the two-sided two-sample t-test, where P1 is the p-value for the comparison with K14E7 Fancd2^-/-^; P2 is the p-value for the comparison with K14E7 Fancd2^+/+^, and P3 is the p-value for the comparison with Fancd2^-/-^.
