# Supplementary material for: Evolution of malignant plasmacytoma cell lines from K14E7 Fancd2−/− mouse long-term bone marrow cultures
Source: Oncotarget. 2016 Sep 15;7(42):68449–72. doi: 10.18632/oncotarget.12036 (PMC5356567; doi:10.18632/oncotarget.12036)
Supplement: Supplementary file 4 [file oncotarget-07-68449-s004.docx]

**Supplemental Table 3: Analysis of non-adherent cells per flask from LTBMCs of a K14E7 Fancd2^-/-^ mice (x 1000000).**

| group | **Week 1** | **Week 2** | **Week 3** | **Week 4** | **Week 5** | **Week 6** |
| --- | --- | --- | --- | --- | --- | --- |
| K14E7 Fancd2 -/- | 1.6±0.2 (n=2) | 0.9±0.1 (n=2) | 1.1±0.1 (n=2) | 1.0±0.2 (n=2) | 0.8±0.1 (n=2) | 0.5±0.4 (n=2) |
| K14E7 Fancd2+/+ | 2.8±0.1 (n=2) p1=0.016 | 2.7±0.0 (n=2) p1=0.0021 | 2.2±0.3 (n=2) p1=0.029 | 2.4±0.3 (n=2) p1=0.022 | 1.5±0.2 (n=2) p1=0.041 | 0.2±0.1 (n=2) p1=0.42 |
| Fancd2-/- | 2.8±0.5 (n=4) p1=0.040  p2=0.96 | 0.8±0.2 (n=4) p1=0.97 p2=0.0002 | 0.7±0.1 (n=4) p1=0.022 p2=0.0006 | 1.1±0.1 (n=4) p1=0.29 p2=0.0008 | 0.9±0.1 (n=4) p1=0.21 p2=0.015 | 1.2±0.4 (n=4) p1=0.13  p2=0.032 |
| Fancd2+/+ | 3.0±0.3 (n=4) p1=0.0051 p2=0.38 p3=0.50 | 2.0±0.3 (n=4) p1=0.0098 p2=0.055 p3=0.0008 | 1.3±0.2 (n=4) p1=0.24 p2=0.0057 p3=0.0021 | 1.8±0.7 (n=4) p1=0.18 p2=0.32  p3=0.14 | 2.1±0.3 (n=4) p1=0.0059 p2=0.080 p3=0.0006 | 3.5±0.2 (n=4) p1=0.0003 p2<0.0001 p3=0.0001 |
| group | **Week 7** | **Week 8** | **Week 9** | **Week 10** | **Week 11** | **Week 12** |
| K14E7 Fancd2 -/- | 0.3±0.0 (n=2) | 0.3±0.1 (n=2) | 0.2±0.0 (n=2) | 0.2±0.0 (n=2) | 0.2±0.1 (n=2) | 0.2±0.1 (n=2) |
| K14E7 Fancd2+/+ | 1.3±0.1 (n=2) p1=0.0047 | 1.5±0.7 (n=2) p1=0.13 | 0.7±0.0 (n=2) p1=0.027 | 0.6±0.1 (n=2) p1=0.050 | 0.4±0.1 (n=2) p1=0.074 | 0.1±0.1 (n=2) p1=0.60 |
| Fancd2 -/- | 0.8±0.2 (n=4) p1=0.030 p2=0.024 | 0.4±0.1 (n=4) p1=0.26 p2=0.27 | 0.4±0.1 (n=4) p1=0.20 p2=0.0072 | 0.2±0.1 (n=3) p1=0.70 p2=0.039 | 0.2±0.1 (n=3) p1=0.48 p2=0.10 | 0.3±0.1 (n=3) p1=0.46  p2=0.27 |
| Fancd2+/+ | 2.4±0.5 (n=4) p1=0.0039 p2=0.035 p3=0.0007 | 1.1±0.3 (n=4) p1=0.013 p2=0.36 p3=0.0027 | 0.7±0.3 (n=4) p1=0.11 p2=0.90 p3=0.073 | 0.6±0.1 (n=4) p1=0.015 p2=0.63 p3=0.014 | 0.4±0.2 (n=4) p1=0.11 p2=0.73 p3=0.10 | 0.4±0.1 (n=3) p1=0.15  p2=0.11  p3=0.27 |
| group | **Week 13** | **Week 14** | **Week 15** | **Week 16** | **Week 17** | **Week 18** |
| K14E7 Fancd2 -/- | 0.2±0.0 (n=2) | 0.2±0.0 (n=2) | 0.2±0.0 (n=2) | 0.2±0.0 (n=2) | 0.2±0.0 (n=2) | 0.2±0.0 (n=2) |
| K14E7 Fancd2+/+ | 0.2±0.0 (n=2) p1=0.048 | 0.2±0.0 (n=2) p1=0.0077 | 1.2±1.3 (n=2) p1=0.47 | 0.3±0.0 (n=2) p1=0.14 | 0.3±0.0 (n=2) p1=0.045 | 0.3±0.1 (n=2) p1=0.25 |
| Fancd2-/- | 0.3±0.1 (n=3) p1=0.28 p2=0.78 | 0.2±0.0 (n=3) p1=0.24 p2=0.024 | 0.2±0.1 (n=2) p1=0.89  p2=0.39 | 0.2±0.0 (n=2) p1=0.66 p2=0.071 | 0.2±0.0 (n=2) p1=0.0068 p2=0.017 | 0.1±0.0 (n=2) p1=0.24  p2=0.15 |
| Fancd2+/+ | 0.4±0.1 (n=3) p1=0.051 p2=0.11 p3=0.14 | 0.3±0.2 (n=3) p1=0.39 p2=0.64 p3=0.39 | 0.5±0.5 (n=2) p1=0.55  p2=0.54 p3=0.48 | 0.3±0.3 (n=2) p1=0.61 p2=0.77 p3=0.58 | 0.2±0.2 (n=2) p1=0.98 p2=0.43 p3=0.66 | 0.3±0.2 (n=2) p1=0.71  p2=0.82  p3=0.48 |
| group | **Week 19** | **Week 20** | **Week 21** |  |  |  |
| K14E7 Fancd2 -/- | No data | No data | No data |  |  |  |
| K14E7 Fancd2+/+ | No data | No data | No data |  |  |  |
| Fancd2-/- | 0.2±0.0 (n=2) | 0.2±0.0 (n=2) | 0.2±0.0 (n=2) |  |  |  |
| Fancd2+/+ | 0.3±0.1 (n=2) p3=0.39 | 0.3±0.0 (n=2) p3=0.023 | 0.3±0.0 (n=2) p3=0.0079 |  |  |  |

Data are summarized with mean + standard deviation, and compared with the two-sided two-sample t-test, where P1 is the p-value for the comparison with K14E7 Fancd2^-/-^; P2 is the p-value for the comparison with K14E7 Fancd2^+/+^; and P3 is the p-value for the comparison with Fancd2^-/-^.
