# Supplementary material for: Evolution of malignant plasmacytoma cell lines from K14E7 Fancd2−/− mouse long-term bone marrow cultures
Source: Oncotarget. 2016 Sep 15;7(42):68449–72. doi: 10.18632/oncotarget.12036 (PMC5356567; doi:10.18632/oncotarget.12036)
Supplement: Supplementary file 5 [file oncotarget-07-68449-s005.docx]

**Supplemental Table 4: Analysis of day 7 colony forming progenitor cells from K14E7 Fancd2^-/-^ LTBMCs.**

| group | **Week 1** | **Week 2** | **Week 3** | **Week 4** | **Week 5** | **Week 6** |
| --- | --- | --- | --- | --- | --- | --- |
| K14E7 Fancd2-/- | 125.3±5.5 (n=3) | 48.3±5.5 (n=3) | 39.3±3.5 (n=3) | 35.7±3.5 (n=3) | 16.0±4.0 (n=3) | 1.0±1.0 (n=3) |
| K14E7 Fancd2+/+ | 128.0±7.5 (n=3)  p1=0.65 | 109.0±5.0 (n=3) p1=0.0001 | 150.0±6.6 (n=3) p1<0.0001 | 76.0±5.0 (n=3) p1=0.0003 | 39.3±3.5 (n=3) p1=0.0016 | 15.7±4.5 (n=3) p1=0.0053 |
| Fancd2 -/- | 88.3±7.1 (n=3) p1=0.0020 p2=0.0027 | 24.7±4.5 (n=3) p1=0.0045 p2<0.0001 | 53.0±4.0 (n=3) p1=0.011 p2<0.0001 | 72.3±4.0 (n=3) p1=0.0003 p2=0.38 | 63.3±3.5 (n=3) p1=0.0001 p2=0.0011 | 8.3±2.1 (n=3) p1=0.0053  p2=0.063 |
| Fancd2+/+ | 120.0±11.5 (n=3)  p1=0.51 p2=0.37 p3=0.016 | 24.3±2.5 (n=3) p1=0.0024 p2<0.0001 p3=0.92 | 133.7±6.0 (n=3) p1<0.0001 p2=0.034 p3<0.0001 | 112.3±3.5 (n=3) p1<0.0001 p2=0.0005 p3=0.0002 | 115.0±5.6 (n=3) p1<0.0001 p2<0.0001 p3=0.0002 | 43.0±4.0 (n=3) p1=0.0001 p2=0.0014 p3=0.0002 |
| group | **Week 7** | **Week 8** | **Week 9** | **Week 10** | **Week 11** | **Week 12** |
| K14E7 Fancd2 -/- | 4.0±1.0 (n=3) | 1.7±0.6 (n=3) | 3.0±1.0 (n=3) | 0.0±0.0 (n=3) | No data | No data |
| K14E7 Fancd2+/+ | 15.0±2.0 (n=3) p1=0.0010 | 26.0±5.0 (n=3) p1=0.013 | 9.3±2.1 (n=3) p1=0.0090 | 21.7±4.0 (n=3) p1=0.011 | 9.3±2.1 (n=3) | 3.7±0.6 (n=3) |
| Fancd2 -/- | 15.0±3.0 (n=3) p1=0.0038 p2=1.00 | 16.0±3.0 (n=3) p1=0.001 p2=0.041 | 3.3±1.2 (n=3) p1=0.72 p2=0.012 | 3.0±1.0 (n=3) p1=0.035 p2=0.0015 | 1.0±1.0 (n=3) p2=0.0033 | 1.0±0.0 (n=3) p2=0.015 |
| Fancd2+/+ | 64.7±4.5 (n=3) p1<0.0001 p2=0.0001 p3=0.0001 | 50.7±4.5 (n=3) p1=0.0025 p2=0.0032 p3=0.0004 | 79.0±7.0 (n=3) p1=0.0024 p2=0.0001 p3=0.0001 | 43.7±4.5 (n=3) p1=0.0035 p2=0.0033 p3=0.0001 | 54.3±5.5 (n=3) p2=0.0002 p3=0.0001 | 73.0±4.0 (n=3) p2=0.0009 p3=0.0010 |
| group | **Week 13** | **Week 14** | **Week 15** | **Week 16** | **Week 17** | **Week 18** |
| K14E7 Fancd2 -/- | No data | No data | No data | No data | No data | No data |
| K14E7 Fancd2+/+ | No data | No data | No data | No data | No data | No data |
| Fancd2 -/- | 0.0±0.0 (n=3) | 0.0±0.0 (n=3) | 0.0±0.0 (n=3) | 0.0±0.0 (n=3) | 0.0±0.0 (n=3) | No data |
| Fancd2+/+ | 45.0±6.0 (n=3) p3=0.0059 | 61.0±6.6 (n=3) p3=0.0038 | 53.0±3.6 (n=3) p3=0.0015 | 18.0±3.0 (n=3) p3=0.0091 | 19.3±2.1 (n=3) p3=0.0038 | 5.0±1.0 (n=3) |
| group | **Week 19** | **Week 20** | **Week 21** |  |  |  |
| K14E7 Fancd2 -/- | No data | No data | No data |  |  |  |
| K14E7 Fancd2+/+ | No data | No data | No data |  |  |  |
| Fancd2 -/- | No data | No data | No data |  |  |  |
| Fancd2+/+ | 0.0±0.0 (n=3) | 0.0±0.0 (n=3) | No data |  |  |  |

Data are summarized with mean + standard deviation, and compared with the two-sided two-sample t-test, where P1 is the p-value for the comparison with K14E7 Fancd2^-/-^; P2 is the p-value for the comparison with K14E7 Fancd2^+/+^; and P3 is the p-value for the comparison with Fancd2^-/-^.
