# Supplementary material for: Evolution of malignant plasmacytoma cell lines from K14E7 Fancd2−/− mouse long-term bone marrow cultures
Source: Oncotarget. 2016 Sep 15;7(42):68449–72. doi: 10.18632/oncotarget.12036 (PMC5356567; doi:10.18632/oncotarget.12036)
Supplement: Supplementary file 6 [file oncotarget-07-68449-s006.docx]

**Supplemental Table 5: Analysis of day 14 colony forming cells from LTBMCs from K14E7 Fancd2^-/-^ mice.**

| group | **Week 1** | **Week 2** | **Week 3** | **Week 4** | **Week 5** | **Week 6** |
| --- | --- | --- | --- | --- | --- | --- |
| K14E7 Fancd2 -/- | 208.3±5.5 (n=3) | 80.7±4.5 (n=3) | 45.3±3.5 (n=3) | 87.0±7.0 (n=3) | 21.7±4.0 (n=3) | 1.7±1.5 (n=3) |
| K14E7 Fancd2+/+ | 306.7±5.0 (n=3) p1<0.0001 | 144.7±4.5 (n=3) p1=0.0001 | 361.3±11.2 (n=3) p1<0.0001 | 383.3±7.8 (n=3) p1<0.0001 | 125.3±6.0 (n=3) p1<0.0001 | 91.0±6.6 (n=3) p1<0.0001 |
| Fancd2 -/- | 120.3±8.0 (n=3) p1=0.0001 p2<0.0001 | 126.3±5.5 (n=3) p1=0.0004 p2=0.011 | 89.0±7.0 (n=3) p1=0.0006 p2<0.0001 | 88.3±4.0 (n=3) p1=0.79 p2<0.0001 | 112.7±6.5 (n=3) p1<0.0001 p2=0.068 | 12.7±1.5 (n=3) p1=0.0009 p2<0.0001 |
| Fancd2+/+ | 299.0±6.6 (n=3) p1=0.0001 p2=0.18 p3<0.0001 | 154.0±3.6 (n=3) p1<0.0001 p2=0.049 p3=0.0019 | 289.7±6.5 (n=3) p1<0.0001 p2=0.0007 p3<0.0001 | 274.0±7.5 (n=3) p1<0.0001 p2=0.0001 p3<0.0001 | 182.7±7.1 (n=3) p1<0.0001 p2=0.0004 p3=0.0002 | 102.0±5.6 (n=3) p1<0.0001 p2=0.091 p3<0.0001 |
| group | **Week 7** | **Week 8** | **Week 9** | **Week 10** | **Week 11** | **Week 12** |
| K14E7 Fancd2 -/- | 5.3±1.5 (n=3) | 2.0±0.0 (n=3) | 3.3±1.2 (n=3) | 1.7±1.2 (n=3) | No data | No data |
| K14E7 Fancd2+/+ | 48.3±6.0 (n=3) p1=0.0003 | 82.7±7.0 (n=3) p1=0.0025 | 59.7±5.5 (n=3) p1=0.0001 | 66.3±5.5 (n=3) p1<0.0001 | 36.0±3.0 (n=3) | 47.7±6.5 (n=3) |
| Fancd2 -/- | 24.3±4.5 (n=3) p1=0.0023 p2=0.0053 | 44.3±4.5 (n=3) p1=0.0038 p2=0.0014 | 20.7±3.5 (n=3) p1=0.0013 p2=0.0005 | 17.0±4.0 (n=3) p1=0.0031 p2=0.0002 | 7.0±2.0 (n=3) p2=0.0002 | 1.7±0.6 (n=3) p2=0.0063 |
| Fancd2+/+ | 187.7±6.5 (n=3) p1<0.0001 p2<0.0001 p3<0.0001 | 155.0±5.6 (n=3) p1=0.0004 p2=0.0002 p3<0.0001 | 234.3±6.1 (n=3) p1<0.0001 p2<0.0001 p3<0.0001 | 163.7±7.5 (n=3) p1=0.0006 p2=0.0001 p3<0.0001 | 188.3±8.0 (n=3) p2<0.0001 p3<0.0001 | 156.0±6.6 (n=3) p2<0.0001 p3=0.0006 |
| group | **Week 13** | **Week 14** | **Week 15** | **Week 16** | **Week 17** | **Week 18** |
| K14E7 Fancd2 -/- | No data | No data | No data | No data | No data | No data |
| K14E7 Fancd2+/+ | 15.3±2.5 (n=3) | 9.0±2.0 (n=3) | 9.0±3.6 (n=3) | 22.3±2.5 (n=3) | No data | No data |
| Fancd2 -/- | 8.7±3.1 (n=3) p2=0.043 | 0.0±0.0 (n=3) p2=0.016 | 0.0±0.0 (n=3) p2=0.050 | 0.0±0.0 (n=3) p2=0.0042 | 0.0±0.0 (n=3) | No data |
| Fancd2+/+ | 162.0±7.5 (n=3) p2<0.0001 p3<0.0001 | 114.7±4.5 (n=3) p2<0.0001 p3=0.0005 | 195.0±6.1 (n=3) p2<0.0001 p3=0.0003 | 60.0±5.6 (n=3) p2=0.0004 p3=0.0029 | 56.3±5.0 (n=3) p3=0.0027 | 15.3±3.5 (n=3) |
| group | **Week 19** | **Week 20** | **Week 21** |  |  |  |
| K14E7 Fancd2 -/- | No data | No data | No data |  |  |  |
| K14E7 Fancd2+/+ | No data | No data | No data |  |  |  |
| Fancd2 -/- | No data | No data | No data |  |  |  |
| Fancd2+/+ | 0.3±0.6 (n=3) | 0.0±0.0 (n=3) | No data |  |  |  |

Data are summarized with mean + standard deviation, and compared with the two-sided two-sample t-test, where P1 is the p-value for the comparison with K14E7 Fancd2^-/-^; P2 is the p-value for the comparison with K14E7 Fancd2^+/+^; and P3 is the p-value for the comparison with Fancd2^-/-^.
